# Perturbation and stability of PAM50 subtyping in population-based primary invasive breast cancer

**DOI:** 10.1038/s41523-023-00589-0

**Published:** 2023-10-19

**Authors:** Srinivas Veerla, Lennart Hohmann, Deborah F. Nacer, Johan Vallon-Christersson, Johan Staaf

**Affiliations:** 1 Division of Oncology, Department of Clinical Sciences, Lund University, Lund, Sweden; 2 Division of Translational Cancer Research, Department of Laboratory Medicine, Lund University, Lund, Sweden

**Keywords:** Breast cancer, Cancer genomics

## Abstract

PAM50 gene expression subtypes represent a cornerstone in the molecular classification of breast cancer and are included in risk prediction models to guide therapy. We aimed to illustrate the impact of included genes and biological processes on subtyping while considering a tumor’s underlying clinical subgroup defined by ER, PR, and HER2 status. To do this we used a population-representative and clinically annotated early-stage breast tumor cohort of 6233 samples profiled by RNA sequencing and applied a perturbation strategy of excluding co-expressed genes (gene sets). We demonstrate how PAM50 nearest-centroid classification depends on biological processes present across, but also within, ER/PR/HER2 subgroups and PAM50 subtypes themselves. Our analysis highlights several key aspects of PAM50 classification. Firstly, we demonstrate the tight connection between a tumor’s nearest and second-nearest PAM50 centroid. Additionally, we show that the second-best subtype is associated with overall survival in ER-positive, HER2-negative, and node-negative disease. We also note that *ERBB2* expression has little impact on PAM50 classification in HER2-positive disease regardless of ER status and that the Basal subtype is highly stable in contrast to the Normal subtype. Improved consciousness of the commonly used PAM50 subtyping scheme will aid in our understanding and interpretation of breast tumors that have seemingly conflicting PAM50 classification when compared to clinical biomarkers. Finally, our study adds further support in challenging the common misconception that PAM50 subtypes are distinct classes by illustrating that PAM50 subtypes in tumors represent a continuum with prognostic implications.

## Introduction

Breast cancer is the most frequent malignancy in women^1^. Today most patients are diagnosed with early-stage breast cancer and are candidates for (neo)adjuvant systemic treatment with curative intent. Treatment decisions and prognostication are routinely based on clinical and pathological assessments of different factors such as menopausal status, disease burden, Nottingham histological grade, and immunohistochemical measurements of estrogen receptor (ER), progesterone receptor (PR), human epidermal growth factor receptor 2 (ERBB2/HER2) (including copy number assessment of *ERBB2* by in situ hybridization), and the proliferation marker protein Ki67^2^. The ER, PR, and ERBB2/HER2 markers also define four major clinical subgroups of breast cancer: (1) ER-positive and HER2-negative tumors (ERpHER2n), (2) ER-positive and HER2-positive tumors (ERpHER2p), (3) ER-negative and HER2-positive (ERnHER2p), and (4) triple-negative breast cancer (TNBC, negative status for ER, PR, and HER2). More recently, multigene expression-based assays have been included in modern treatment guidelines based on evidence that they can aid particularly in selecting patients with ERpHER2n disease that benefit from adjuvant chemotherapy in addition to endocrine treatment^3–5^. Clinical use of such gene expression-based signatures has largely been restricted to commercial implementations using targeted assays^6^.

An example of a clinical multigene test is the Prosigna assay, which uses the nearest-centroid classification for PAM50 molecular subtype assignment^7^. The PAM50 classification scheme by Parker et al.^7^ builds upon the seminal work by Perou et al.^8^ and defines five molecular subtypes in breast cancer: (1) basal-like (Basal), (2) HER2-enriched (HER2E), (3) luminal A (LumA), (4) luminal B (LumB), and (5) normal-like (Normal). The prognostic value of these molecular subtypes has repeatedly been demonstrated^9–14^. The subtypes are associated with specific transcriptional patterns that may also be interpreted as molecular processes including, e.g., low expression of ER-status-related genes (such as *GATA3*, *CA12*, *XBP1*, and *FOXA1* in Basal tumors^15^), high expression of basal cell keratins (e.g., *KRT5*, *KRT14*, and *KRT17* in Basal and Normal subtypes^15^), high expression of genes in the 17q12 amplicon (*ERBB2*/*GRB7*) in *ERBB2*-amplified tumors, and overall higher expression of proliferation-related genes in LumB, HER2E, and Basal subtypes compared to mainly LumA tumors^7^. Importantly, the PAM50 gene centroid values used for subtyping new samples reflect these transcriptional patterns/molecular processes in the samples (cohort) from which they were originally derived. Typical PAM50 subtyping includes measuring the distance (usually correlation-based distance) in relative gene expression space from a sample to the reported PAM50 subtype centroids and selecting the nearest one (highest correlation), i.e., nearest-centroid (NC) classification. The need for relative gene expression for subtyping typically requires normalization to transform gene expression values of samples to be subtyped relative to a reference. This step is important as inadequate normalization can result in erroneous classification^16–21^. Consequently, single sample predictors based on, e.g., gene rules have been reported recently to try to circumvent this issue^14,16^.

Specific PAM50 subtypes have been shown to be enriched in different clinical subgroups of breast cancer, with the respective characteristic association of the Basal subtype with TNBC, the HER2E subtype with ERnHER2p tumors, and the LumA and LumB subtypes with the ERpHER2n clinical subgroup (see ref. ^22^). Still, less typical subtype patterns appear when applying the PAM50 NC classification to large population-representative cohorts, as shown by Vallon-Christersson et al.^22^. In this large study of >3500 population-representative primary breast cancers profiled by RNA sequencing, it was observed that essentially all subtypes are represented, albeit often in small proportions, in clinical treatment groups defined by the combination of ER, PR, and HER2 status and the administered therapy. This observation is expected based on the nature of NC classification and its dependency on gene centering as shown in both breast and lung cancer^16,20^. Nevertheless, it raises the question of how to interpret for instance a clinically defined ERpHER2n tumor classified as PAM50 Basal or HER2E, or a TNBC tumor classified as LumA or LumB. For such tumors, it may be questioned whether PAM50 subtypes are clinically or molecularly relevant, if they merely reflect the nature of NC classification, or if other biological processes and gene expression patterns that correlate with the original prototypical subtype samples come into play. In addition, while the Risk Of Recurrence (ROR) score—a read-out of the commercial Prosigna test and used for risk stratification of patients—includes additional components related to tumor size and tumor proliferation, PAM50 subtype correlations contribute a major part of the final score^7^.

In the present study, we wanted to investigate which biological processes/genes represented in the PAM50 centroids drive tumor subtyping while considering the clinical subgroups: TNBC, ERnHER2p, ERpHER2p, ERpHER2n, and if these impact the prognostic association of PAM50 subtypes. The rationale behind this investigation is that the processes and genes represented in PAM50 may have different influences on subtyping depending on the clinical subgroup, which may explain unexpected results such as ERpHER2n tumors classified as PAM50 Basal. To achieve this, we used a recently reported population-based cohort of uniformly accrued early-stage primary breast cancers comprising 6233 patients analyzed by whole transcriptome RNA sequencing with available PAM50 NC subtypes and a presented rigorous NC classification strategy^14^. We illustrate that PAM50 subtyping is dependent on different biological processes in different clinical breast cancer subgroups, but also within subgroups and PAM50 subtypes themselves. In combination with the hardcoded interrelationship of gene expression patterns in the actual PAM50 centroids themselves, these factors can account for the observation of subtypes conceptually not in line with clinical marker-defined subgroups.

In the end, an improved understanding of the PAM50 subtyping scheme may aid in the interpretation of tumors with seemingly disparate molecular-clinical classifications. Moreover, the results presented in this study further illustrate that PAM50 subtypes in breast cancer represent a continuum rather than distinct classes, and acknowledging this has prognostic implications.

## Results

### PAM50_NC_ classification correlation strength and second-best subtype vary between clinical subgroups

**Fig. 1:**
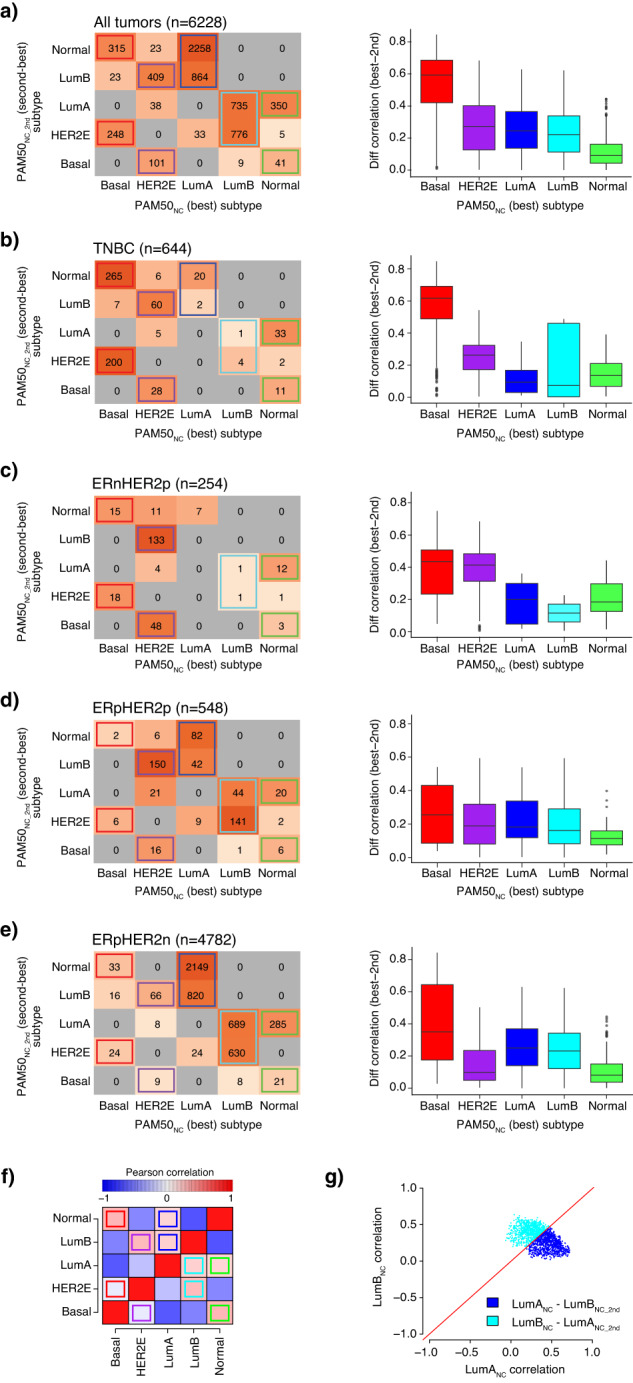
Patterns of PAM50_NC_ versus PAM50_NC_2nd_ subtype. In panels (**a**–**e**), the left panels show the cross-tabulated PAM50_NC_ subtype versus the PAM50_NC_2nd_ subtype for separate tumor subsets, whereas the right panels show the corresponding difference (delta) in Spearman correlation between PAM50_NC_ and PAM50_NC_2nd_ subtype based on the average Spearman correlation of the 100 NC classifications for each case. In the cross tables, colored boxes highlight consistent subtype patterns between PAM50_NC_ and PAM50_NC_2nd_ subtypes. Of all 6233 tumors, 6228 had an unambiguous second-best subtype based on NC classification. **a** All SCAN-B tumors. **b** TNBC tumors. **c** ERnHER2p tumors. **d** ERpHER2p tumors. **e** ERpHER2n tumors. **f** Heatmap of Pearson correlations between PAM50 centroids. Heatmap cells marked with colored boxes show centroid correlation patterns consistent with the PAM50_NC_ and PAM50_NC_2nd_ subtype patterns shown in panels (**a**–**e**). **g** Scatter plot of LumA correlation values versus LumB correlation values for tumors classified as LumA_NC_ – LumB_NC_2nd_ or LumB_NC_ – LumA_NC_2nd_ (*n* = 1599). The red line corresponds to a 1:1 relationship between correlation estimates. Boxplot elements correspond to: (1) center line = median, (2) box limits = upper and lower quartiles, (3) whiskers = 1.5x interquartile range.

In the total cohort of 6233 tumors, 645 were TNBC (10.3%), 254 ERnHER2p (4.1%), 548 ERpHER2p (8.8%), and 4786 ERpHER2n (76.8%). PAM50_NC_ subtype proportions in these clinical subgroups were: (1) TNBC—73.3% Basal_NC_, 15.4% HER2E_NC_, 3.4% LumA_NC_, 0.8% LumB_NC_, and 7.1% Normal_NC_, (2) ERnHER2p—13.0% Basal_NC_, 77.2% HER2E_NC_, 2.8% LumA_NC_, 0.8% LumB_NC_, and 6.3% Normal_NC_, (3) ERpHER2p—1.5% Basal_NC_, 35.2% HER2E_NC_, 24.3% LumA_NC_, 34.0% LumB_NC_, and 5.1% Normal_NC_, and (4) ERpHER2n—1.5% Basal_NC_, 1.8% HER2E_NC_, 62.6% LumA_NC_, 27.8% LumB_NC_, and 6.4% Normal_NC_. In PAM50 classification the subtype assigned to a tumor sample is typically the subtype centroid with the highest correlation (NC classification) to that sample. Consequently, there is always a second-best PAM50 subtype (PAM50_NC_2nd_) as correlation is computed for all centroids. To investigate the relationship between PAM50_NC_ and PAM50_NC_2nd_ subtypes in primary invasive breast cancer, we cross-tabulated subtypes for the complete cohort as well as separately for the TNBC, ERnHER2p, ERpHER2p, and ERpHER2n groups (Fig. 1a–e). A consistent pattern was observed across all tumor groups. Briefly, the PAM50_NC_2nd_ subtype for Basal_NC_ tumors was mainly HER2E_NC_2nd_ or Normal_NC_2nd_, for HER2E_NC_ tumors mainly LumB_NC_2nd_ or Basal_NC_2nd_, for LumA_NC_ mainly LumB_NC_2nd_ or Normal_NC_2nd_, for LumB_NC_ mainly LumA_NC_2nd_ or HER2E_NC_2nd_, and for Normal_NC_ tumors mainly LumA_NC_2nd_ or Basal_NC_2nd_. This pattern corresponded perfectly to positive correlation patterns between PAM50 centroids (Fig. 1f). Next, we investigated the difference in Spearman correlation (delta) between the best (PAM50_NC_) and second-best (PAM50_NC_2nd_) subtype for all tumors as well as separately for the four clinical subgroups, as it represents to some extent a measurement of how distinct a sample’s best subtype is from its second-best alternative (boxplots in Fig. 1a–e). Overall, the Basal_NC_ subtype showed the largest delta in the correlation between PAM50_NC_ and PAM50_NC_2nd_, while the Normal_NC_ subtype generally showed the smallest. In the ERnHER2p subgroup, but also somewhat in ERpHER2p tumors, the HER2E_NC_ subtype showed a larger delta. This is consistent with especially ERnHER2p tumors being generally considered to have a strong overrepresentation of the HER2E_NC_ subtype. Still, there is a clear difference in the distinctiveness of HER2E_NC_ correlation in HER2-positive tumors depending on ER status (regarding both the actual delta and the distribution/tightness of these values). Specifically, ERnHER2p HER2E_NC_ tumors showed larger delta between the PAM50_NC_ and PAM50_NC_2nd_ subtype compared to ERpHER2p HER2E_NC_ tumors. LumA_NC_ and LumB_NC_ subtypes appeared to have equal differences within tested groups. Here it should be noted that despite the discrete subtype assignment of a tumor in these analyses, it is still evident from the actual centroid correlation values that LumA_NC_ vs. LumB_NC_ subtyping represents a continuum rather than distinct subsets of samples, as illustrated in Fig. 1g for the 1599 tumors labeled LumA_NC_ – LumB_NC_2nd_ and LumB_NC_ – LumA_NC_2nd_.

### Evaluating the prognostic value of the second-best PAM50 subtype in adjuvant endocrine-treated lymph node-negative ERpHER2n patients

**Fig. 2:**
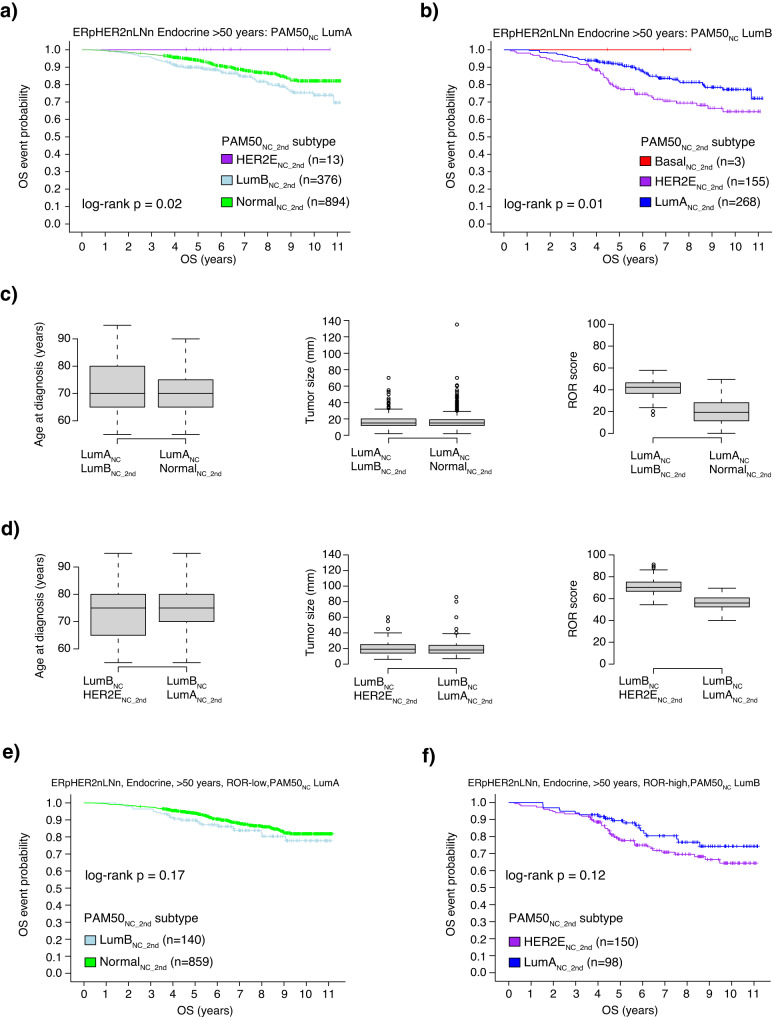
Association of PAM50_NC_2nd_ subtype with patient outcome. **a** Overall survival (OS) for endocrine-treated ERpHER2nLNn patients >50 years of age that were PAM50_NC_ subtyped as LumA_NC_. Patients are stratified by their PAM50_NC_2nd_ subtype. **b** Overall survival for endocrine-treated ERpHER2nLNn patients >50 years of age that were PAM50_NC_ subtyped as LumB_NC_. Patients are stratified by their PAM50_NC_2nd_ subtype. **c** Distributions for age at diagnosis (left), tumor size (center), and ROR T0 scores (right) obtained from ref. ^14^ in endocrine-treated ERpHER2nLNn patients >50 years of age comparing cases subtyped as LumA_NC_ – LumB_NC_2nd_ versus LumA_NC_ – Normal_NC_2nd_. **d** Distributions for age at diagnosis (left), tumor size (center), and ROR T0 scores (right) obtained from ref. ^14^ in endocrine-treated ERpHER2nLNn patients >50 years of age comparing cases subtyped as LumB_NC_ – HER2E_NC_2nd_ versus LumB_NC_ – LumA_NC_2nd_. **e** Overall survival for endocrine-treated ERpHER2nLNn patients >50 years of age that were PAM50_NC_ subtyped as LumA_NC_ and as ROR-low risk category according to ref. ^14^. Patients are stratified by their PAM50_NC_2nd_ subtype. **f** Overall survival for endocrine-treated ERpHER2nLNn patients >50 years of age that were PAM50_NC_ subtyped as LumB_NC_ and as ROR-high risk category according to ref. ^14^. Patients are stratified by their PAM50_NC_2nd_ subtype. Boxplot elements correspond to: (1) center line = median, (2) box limits = upper and lower quartiles, (3) whiskers = 1.5x interquartile range.

Next, we set out to determine if the PAM50_NC_2nd_ subtype was of prognostic value. To this end, we focused analyses on adjuvant endocrine-treated lymph node-negative ERpHER2n patients (ERpHER2nLNn) older than 50 years at diagnosis, similar to ref. ^14^. This patient subgroup is typically a main target for gene signature testing as recommended by St Gallen panelists^23^. Using overall survival as a clinical endpoint we observed that a LumA_NC_ – LumB_NC_2nd_ phenotype showed a poorer prognosis compared to the LumA_NC_ – Normal_NC_2nd_ group (Fig. 2a). For PAM50_NC_ tumors subtyped as LumB_NC_, we found that the LumB_NC_ – LumA_NC_2nd_ patient group showed better prognosis compared to LumB_NC_ – HER2E_NC_2nd_ group (Fig. 2b). For distant recurrence-free interval as clinical endpoint there was, however, no statistical differences in prognosis for neither LumA_NC_ nor LumB_NC_ tumors when stratified similarly (log-rank *p* > 0.05 for both comparisons). To further investigate the causes of the overall survival differences we compared patient age and tumor size between the groups and the frequency of lobular histology, ROR scores, and proliferation metagene scores (Fig. 2c, d). In the LumA_NC_ – Normal_NC_2nd_ group, 25% of tumors were of lobular type compared to 11% in the LumA_NC_ – LumB_NC_2nd_ group (Fisher’s exact test, *p* < 0.0001). For LumB_NC_ – LumA_NC_2nd_ tumors and LumB_NC_ – HER2E_NC_2nd_ tumors lobular histology percentages were 10.4% and 8.4%, respectively (Fisher’s exact test *p* > 0.05). Consistent with a better outcome, LumA_NC_ – Normal_NC_2nd_ tumors showed lower ROR scores than LumA_NC_ – LumB_NC_2nd_ tumors (Fig. 2c), and the former group was drastically enriched for ROR-low risk categorized tumors based on data from ref. ^14^ (Fisher’s exact *p* = 2e-117). The ROR-score patterns may be expected as the PAM50 subtype is one component in the ROR-score calculation. However, the ROR-score patterns were mimicked by comparisons of proliferation metagene scores (mitotic checkpoint), where LumA_NC_ – LumB_NC_2nd_ showed significantly higher scores than LumA_NC_ – Normal_NC_2nd_ tumors (Wilcoxon’s test *p* = 3e-39). Consistent patterns for the proliferation metagene were also observed in LumB_NC_ – LumA_NC_2nd_ (lower) versus LumB_NC_ – HER2E_NC_2nd_ (higher) tumors (Wilcoxon’s test *p* = 4e-33). No statistical differences were observed in tumor size between the LumA_NC_ – LumB_NC_2nd_ group and the LumA_NC_ – Normal_NC_2nd_ group (Wilcoxon’s test *p* = 0.06), nor between the LumB_NC_ – LumA_NC_2nd_ group and the LumB_NC_ – HER2E_NC_2nd_ group (Wilcoxon’s test *p* = 0.52) (Fig. 2c, d). No difference was observed for patient age at diagnosis between the LumB_NC_ – LumA_NC_2nd_ versus LumB_NC_ – HER2E_NC_2nd_ group (Wilcoxon’s test *p* = 0.43) (Fig. 2d). While there was a statistically significant difference in age at diagnosis between the LumA_NC_ – LumB_NC_2nd_ versus LumA_NC_ – Normal_NC_2nd_ group (Wilcoxon’s test *p* < 0.001) it should be noted that the two groups had similar median age (70 years) (Fig. 2c). Thus, the statistical significance between distributions might be due to the large sample sizes compared.

In addition, we analyzed whether the LumA_NC_ – Normal_NC_2nd_ vs LumA_NC_ – LumB_NC_2nd_ phenotypes differed with respect to patient outcome within an ROR risk category (low, intermediate, high as determined in ref. ^14^ based on RNA sequencing data). While not reaching statistical significance, we did observe that in the ROR-low group of ERpHER2nLNn adjuvant endocrine-treated group of patients >50 years at diagnosis, patients with LumA_NC_ – Normal_NC_2nd_ tumors showed a trend toward better overall survival (log-rank *p* = 0.17, Fig. 2e). In ROR-high tumors, patients with LumB_NC_ – LumA_NC_2nd_ tumors showed a non-significant trend toward better overall survival compared to patients with tumors subtyped as LumB_NC_ – HER2E_NC_2nd_ (log-rank *p* = 0.12, Fig. 2f). We also compared the inverse, whether ROR groups were associated with outcome within LumA_NC_ – LumB_NC_2nd_ patients or within LumA_NC_ – Normal_NC_2nd_ patients separately, however no such associations were observed (log-rank *p* = 0.6 and *p* = 0.98, respectively).

### Overall impact of the leave-oneGeneCluster-out centroid perturbation strategy on PAM50 classification

**Fig. 3:**
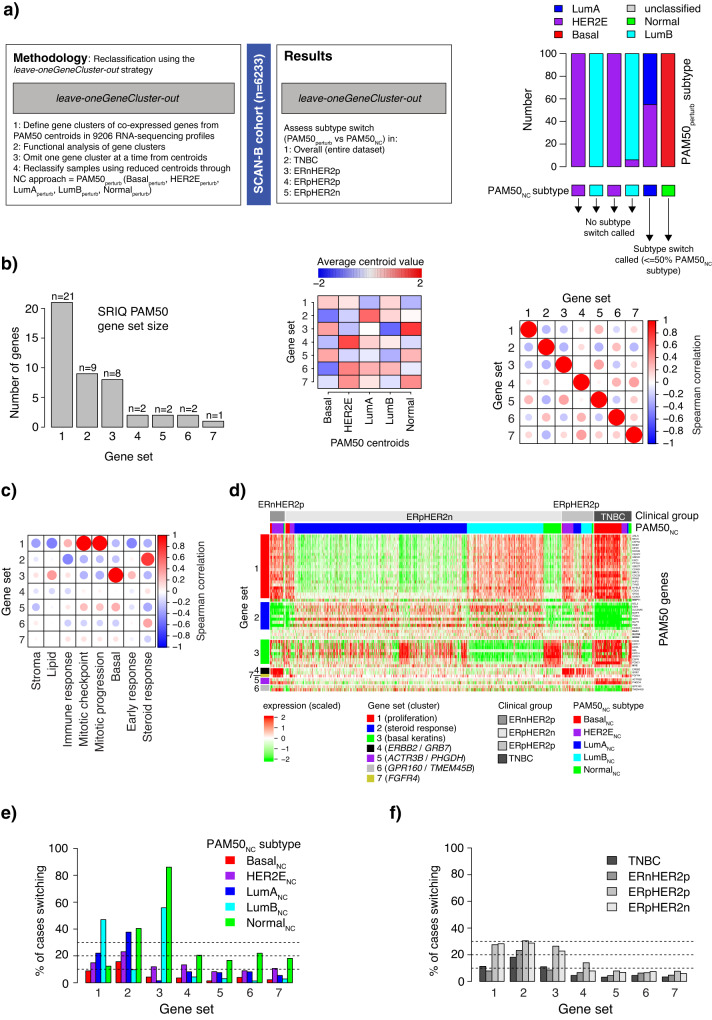
Study overview and PAM50 reclassification results for the *leave-oneGeneCluster-out* strategy. **a** Study outline, perturbation methodology, and subtype switch concept. A sample is called as having a subtype switch if the PAM50_NC_ subtype is observed in ≤50% of the 100 PAM50_perturb_ reclassifications (right panel). **b** Left panel, size of identified SRIQ core gene clusters defined from 9206 RNA sequencing profiles from ref. ^14^. Center panel, heatmap of average PAM50 centroid value for each gene set for each PAM50 centroid subtype. Right panel, Spearman correlation of average SRIQ FPKM gene cluster expression for each gene set combination in all 9206 RNA sequencing profiles. **c** Spearman correlation matrix of average SRIQ FPKM gene cluster expression versus rank-based scores for eight reported biological metagenes from Fredlund et al.^15^ for the 6233 tumors included in this study. **d** Heatmap of scaled FPKM expression for PAM50 genes stratified by SRIQ gene cluster definition and ordered by clinical group and PAM50_NC_ subtype for the 6233 included tumors. **e** Percent of tumors switching subtype (i.e., a different PAM50_perturb_ subtype compared to PAM50_NC_) by the *leave-oneGeneCluster-out* strategy on a whole cohort level stratified by PAM50_NC_ subtypes for the 6233 included tumors. **f** Percent of tumors switching subtype by the *leave-oneGeneCluster-out* strategy on a whole cohort level stratified by tumors’ ER, PR, and HER2 status.

Considering the different patterns of PAM50_NC_ versus PAM50_NC_2nd_ subtype across the four ER and HER2 defined subgroups, we next investigated what happened to PAM50 classification when applying a *leave-oneGeneCluster-out* perturbation strategy where groups of co-expressed genes (gene sets) were excluded and samples reclassified based on the remaining values. The PAM50 reclassification overview and the methodology for this strategy are presented in Fig. 3a. We first utilized SRIQ clustering of the 50 PAM50 genes in 9206 SCAN-B RNA sequencing profiles to define seven core gene clusters of varying size (Fig. 3b, Supplementary Table 2).

Correlations of the average expression for each gene set (gene set scores) with eight proposed biological metagenes in breast cancer^15^ showed that three of the gene sets were strongly correlated with different described metagenes. Gene set 1 (proliferation) was correlated to proliferation metagenes and includes, e.g., *MKI67*, *CENPF*, *CCNE1*, *TYMS*, and *KIF2C*. Gene set 2 (steroid response) was correlated to the steroid response metagene and includes *ESR1*, *FOXA1*, *PGR*, *BCL2*, *SLC39A6*, *MAPT*, *NAT1*, *MLPH*, and *CXXC5*. Gene set 3 (basal keratins) was correlated to a basal cell metagene and included *CDH3*, *KRT17*, *KRT5*, *KRT14*, *MIA*, *SFRP1*, *EGFR*, and *FOXC1* (Fig. 3c). Gene sets 1–3 included enough genes for meaningful functional enrichment analysis that confirmed metagene associations for gene sets 1 and 2 (Supplementary Table 2).

In support of the above analyses, an expression heatmap of the 6233 samples ordered by the four clinical subgroups (TNBC, ERnHER2p, ERpHER2p, ERpHER2n) followed by PAM50_NC_ subtype and genes grouped by the seven gene clusters clearly showed gene cluster co-expression and distinct transcriptional differences (low/high expression) across both clinical subgroups and PAM50_NC_ subtypes (Fig. 3d). Moreover, patterns of individual gene set scores across PAM50_NC_ subtypes in TNBC, ERnHER2p, ERpHER2p, and ERpHER2n tumors further illustrate the association of specific gene sets with different PAM50_NC_ subtypes (Supplementary Fig. 1). Performing a *leave-oneGeneCluster-out* reclassification strategy on the complete cohort (*n* = 6233) showed that excluding specific gene sets had different impact on the proportion of samples switching subtype depending on the PAM50_NC_ subtype (Fig. 3e) or clinical subgroup (Fig. 3f). The largest effects for the Normal_NC_ subtype were observed when excluding gene set 2 (steroid response, ~40% switching) or gene set 3 (basal keratins, ~80% switching). For the LumB_NC_ subtype, the largest effect was seen after the exclusion of gene set 1 (proliferation) or 3 (basal keratins), both causing >40% of tumors to switch subtype. For the LumA_NC_ subtype, the greatest effect was observed when excluding gene set 2 (steroid response), while the proportions of samples switching subtype in the HER2E_NC_ and Basal_NC_ subtypes were comparably lower, with the Basal_NC_ subtype showing highest stability in line with the high subtype distinctiveness as shown in Fig. 1a–e. Interestingly, excluding gene set 4 (*ERBB2*/*GRB7*) caused only a moderate proportion (<15%) of all HER2E_NC_ tumors to change subtype, less than excluding either gene sets 1 or 2. For the clinical subgroups as a whole, the largest perturbation effects were observed for gene sets 1–3 in mainly ER-positive groups (Fig. 3f).

### Impact of the leave-oneGeneCluster-out centroid perturbation strategy on PAM50 classification when stratified by molecular and clinical subgroups

**Fig. 4:**
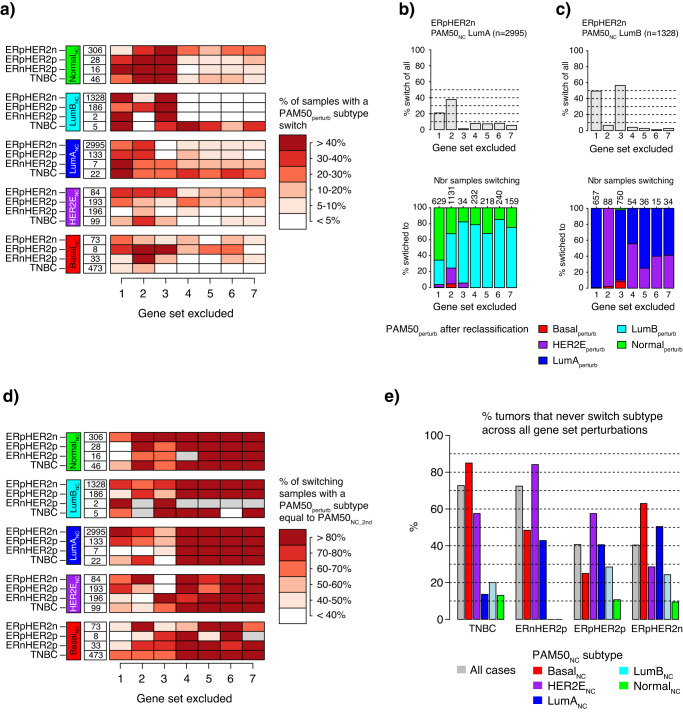
PAM50 reclassification results for the *leave-oneGeneCluster-out* strategy when stratified for molecular and clinical subgroup. **a** Heatmap showing the proportion of tumors that switched subtype after gene set exclusion stratified by molecular and clinical subgroup. Numbers represent total group sizes per row. **b** Top panel shows the proportion of ERpHER2n LumA_NC_ tumors with a PAM50_perturb_ subtype different from their PAM50_NC_ subtype, i.e., switching subtype, when excluding a specific gene set in *leave-oneGeneCluster-out* reclassification. Lower panel shows the distribution of the PAM50_perturb_ subtypes in tumors that switched in the top panel, numbers on top represent the total number of samples that switched subtype. **c** The same illustration as in (**b**), but for ERpHER2n LumB_NC_ tumors. **d** Heatmap showing the proportion of tumors that switched subtype after gene set exclusion that had a PAM50_perturb_ subtype similar to the PAM50_NC_2nd_ subtype. Numbers represent total group sizes per row. **e** Summary bar plots of the percentage of tumors in each clinical group further stratified by their PAM50_NC_ subtype that never switch subtype across all gene set perturbations, i.e., the PAM50_perturb_ subtype is the same as the PAM50_NC_ subtype in all perturbations. These cases are hereon referred to as K0 cases.

We next aimed to address whether the effect of the *leave-oneGeneCluster-out* strategy was different for PAM50_NC_ subtypes within specific clinical subgroups (e.g., TNBC tumors classified as Basal_NC_). To address this question, we evaluated its impact separately for each clinical subgroup (TNBC, ERnHER2p, ERpHER2p, and ERpHER2n) stratified by the PAM50_NC_ subtype. A summary overview of the key results is provided in Fig. 4, with detailed plots for each clinical subgroup provided in Supplementary Figs. 2–5. In addition, we also for each gene set perturbation and combination of PAM50_NC_ and clinical group computed the delta in correlation before and after *leave-oneGeneCluster-out* reclassification (PAM50_NC_ subtype vs. PAM50_perturb_ subtype) (Supplementary Figs. 6–9). Generally, the correlation delta between the subtype calls varied depending on: (1) the excluded gene set, (2) the PAM50_NC_ subtype, and (3) if a tumor switched or not, resulting in both negative and positive correlation differences. This suggests varying importance/relevance of gene sets for classification, although it should be noted that the median difference in Spearman correlation values between PAM50_NC_ versus PAM50_perturb_ values was for most comparisons small (e.g., <±0.2), and that for many comparisons the number of tumors per group is low.

Several key findings are illustrated in the heatmap of PAM50_perturb_ subtype switch proportions (Fig. 4a). Overall, the gene sets appear to affect PAM50_NC_ and clinical groups differently, with both high and low switch proportions inferred depending on subtype, clinical group, and gene set excluded. One observation was that the prototypical Basal_NC_ subtype in TNBC, and the prototypical HER2E_NC_ subtype in ERnHER2p tumors remained stable with <10–15% of tumors switching for all gene set exclusions (Supplementary Figs. 2 and 3 for details). Moreover, we observe that for the smaller gene sets (gene sets 4–7) the proportion of tumors switching subtype is typically low, indicating a relatively low impact on PAM50 classification stability if these genes are present/absent. This observation is especially interesting for gene sets 4 (17q12 amplicon: *ERBB2*/*GRB7*) and 7 (*FGFR4*) that both include genes specifically selected for overexpression in HER2E tumors in the original PAM50 centroids^7^ (see also Supplementary Fig. 1). The largest impact of excluding gene set 4 was seen in ERpHER2p tumors subtyped as HER2E_NC_, with 25.9% of tumors affected in the PAM50_perturb_ classification (Supplementary Fig. 4). In contrast, when gene set 4 and 7 was excluded in ERnHER2p, only 7.7% and 3.6%, respectively of HER2E_NC_ tumors were affected (Supplementary Fig. 3).

Another observation from Fig. 4a is the sensitivity of the Normal_NC_ subtype to the exclusion of gene set 3 (basal keratins) irrespective of the clinical subgroup. Exclusion of this gene set caused a high proportion of Normal_NC_ tumors (up to 80%) to switch indicating the importance of this expression module for the subtyping (see also Supplementary Figs. 2–5 for details and exact proportions). Finally, we observe a strong effect of gene set 1 (proliferation) and gene set 3 (basal keratins) exclusion in LumB_NC_ tumors across clinical groups, somewhat in contrast to LumA_NC_ (Supplementary Figs. 2–5 for details). These observations are further exemplified in detail in Fig. 4b, c for ERpHER2n tumors, also showing to which PAM50_perturb_ subtype a tumor switches when excluding the specific gene sets in the respective group.

Next, we asked how often a tumor that switched subtype did so to a PAM50_perturb_ subtype similar to its PAM50_NC_2nd_ subtype across clinical and molecular groups. The heatmap in Fig. 4d summarizes the results for this question (details are provided in Supplementary Figs. 2–5), showing that for the smaller gene sets PAM50_perturb_ subtypes in tumors switching were most often of the same label as PAM50_NC_2nd_ across subgroups. For the larger gene sets the proportions varied more but were still most often >50%.

Finally, we also investigated the proportion of tumors in the molecular and clinical subgroups that were never affected by the *leave-oneGeneCluster-out* perturbations. As seen in Fig. 4e, the Basal_NC_ subtype in TNBC and the HER2E_NC_ subtype in ERnHER2p tumors were particularly stable in that >80% of tumors never switched subtype irrespective of perturbation. In contrast, the Normal_NC_ was particularly unstable with very low number of completely unaffected tumors, followed by LumB_NC_ and LumA_NC_.

### Impact of the leave-oneGeneCluster-out strategy on PAM50 classification with respect to prognosis

**Fig. 5:**
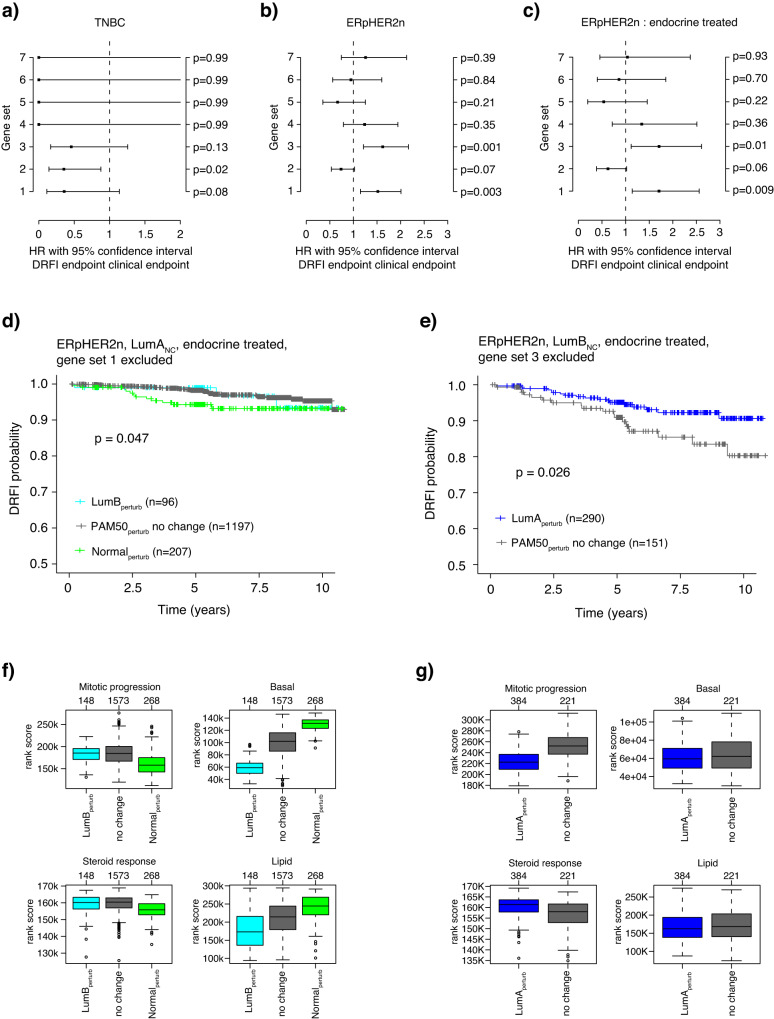
*Leave-oneGeneCluster-out* perturbation and association to patient outcome. Forest plot of hazard ratios with 95% confidence intervals from univariate Cox regression, using DRFI as clinical endpoint, for tumors that switched subtype versus tumors that did not switch subtype (reference) after exclusion of a gene set in **a** TNBC tumors, **b** ERpHER2n tumors, and **c** endocrine-treated ERpHER2n tumors only. **d** Kaplan–Meier plot of DRFI for PAM50_perturb_ subtypes in endocrine-treated ERpHER2n LumA_NC_ tumors after exclusion of gene set 1 (proliferation). **e** Kaplan–Meier plot of DRFI for PAM50_perturb_ subtypes in endocrine-treated ERpHER2n LumB_NC_ tumors after exclusion of gene set 3 (basal keratins). **f** Boxplots of rank-based scores for the mitotic progression, basal, steroid response, and lipid metagenes for endocrine-treated ERpHER2n LumA_NC_ tumors in panel (**d**). **g** Boxplots of rank-based scores for the mitotic progression, basal, steroid response, and lipid metagenes for endocrine-treated ERpHER2n LumB_NC_ tumors in panel (**e**). Note that not all included cases in the study have DRFI outcome data, thus the difference in sample numbers between boxplots and survival plots. Boxplot elements correspond to: (1) center line = median, (2) box limits = upper and lower quartiles, (3) whiskers = 1.5x interquartile range.

To investigate whether the *leave-oneGeneCluster-out* reclassification had any impact on the patient outcome we performed univariate Cox regression (using DRFI as clinical endpoint) for each gene set perturbation in each clinical subgroup, using tumors that were not affected by a perturbation as a reference in the model. In the ERnHER2p and ERpHER2p groups, there were no statistically significant associations for any gene set, whereas in the TNBC, ERpHER2n, and endocrine-treated ERpHER2n groups significant hazard ratios were observed for gene sets 1–3 (Fig. 5a–c). We further stratified endocrine-treated ERpHER2n tumors into LumA_NC_ and LumB_NC_ to illustrate the prognostic associations of the PAM50_perturb_ subtypes in these specific subgroups when excluding gene set 1 (proliferation) in LumA_NC_ (Fig. 5d) or gene set 3 (basal keratins) in LumB_NC_ tumors (Fig. 5e). Importantly, the PAM50_perturb_ subtypes in these two endocrine-treated tumor groups displayed biological metagene rank scores related to proliferation, steroid response, and basal keratins in line with what would be expected when compared to tumors that did not change subtype (Fig. 5f, g).

### Samples that were stable during leave-oneGeneCluster-out perturbation as basis for a refined true single sample PAM50 subtype classification in ERpHER2n tumors

**Fig. 6:**
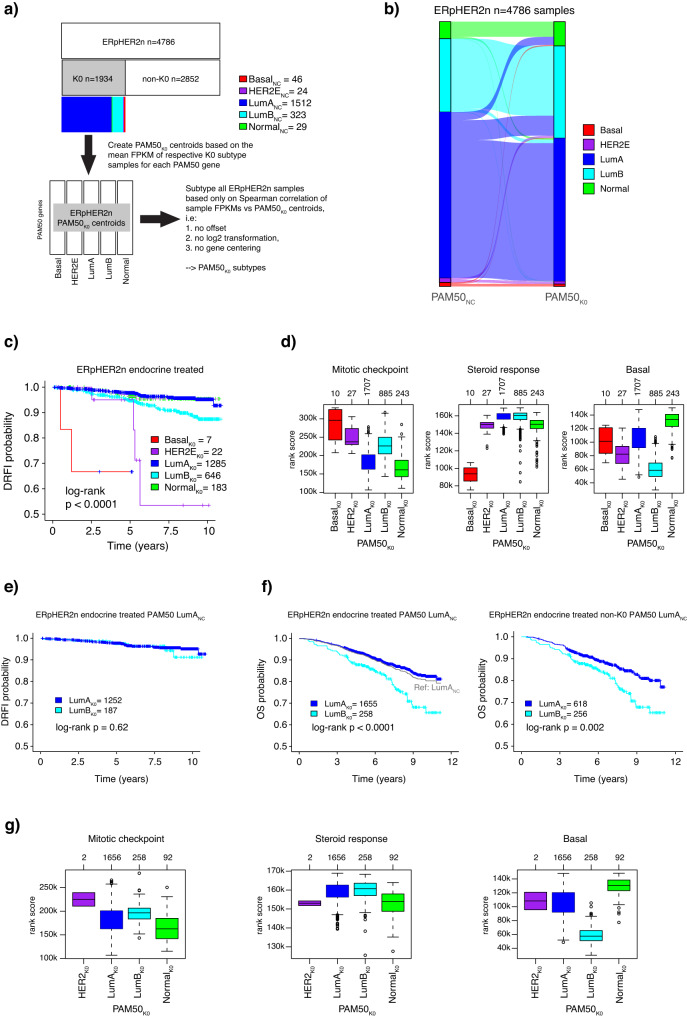
Refined single sample PAM50 subtyping in ERpHER2n tumors based on *leave-oneGeneCluster-out* perturbation stable tumors. **a** Outline of the scheme to create refined ERpHER2n PAM50 centroids (termed PAM50_K0_) used for single sample classification by Spearman correlation based on FPKM values only (i.e., no gene centering). **b** Sankey plot of subtype change for ERpHER2n tumors when performing PAM50_K0_ classification as outlined in (**a**). **c** Kaplan–Meier plot of DRFI for PAM50_K0_ subtypes in endocrine-treated ERpHER2n tumors. **d** Boxplots of rank-based scores for the mitotic checkpoint, steroid response, and basal metagenes for endocrine-treated ERpHER2n tumors stratified by PAM50_K0_ subtypes. **e** Kaplan–Meier plot of DRFI for PAM50_K0_ subtypes in endocrine-treated ERpHER2n LumA_NC_ tumors. HER2E_K0_ and Normal_K0_ groups excluded due to size. **f** Left panel, Kaplan–Meier plot of OS for PAM50_K0_ subtypes in all endocrine-treated ERpHER2n LumA_NC_ tumors. HER2E_K0_ and Normal_K0_ groups excluded due to size. Right panel, same plot but only for non-K0 tumors (i.e., tumors not included in the PAM50_K0_ centroid creation). **g** Boxplots of rank-based scores for the mitotic checkpoint, steroid response, and basal metagenes for endocrine-treated ERpHER2n LumA_NC_ tumors stratified by PAM50_K0_ subtypes. Note that not all included cases in the study have DRFI outcome data, thus the difference in sample numbers between boxplots and survival plots. Boxplot elements correspond to: (1) center line = median, (2) box limits = upper and lower quartiles, (3) whiskers = 1.5x interquartile range.

Considering the impact of PAM50_perturb_ subtype switches on patient outcome shown in Fig. 5, we next hypothesized that *leave-oneGeneCluster-out* perturbation stable tumors (portrayed in Fig. 4e) could represent core PAM50 subtype cases within each clinical group and used to refine PAM50 subtyping in a way that could also affect prognostic associations. To test this hypothesis, we focused on the largest clinical group, ERpHER2n tumors (*n* = 4786). As outlined in Fig. 6a, we first identified the 1934 ERpHER2n tumors that never switched subtype in any gene set perturbation (referred to as K0 tumors). Based on these tumors’ PAM50_NC_ subtypes, we created new centroid values for each PAM50 gene using mean FPKM across tumors in respective subtype, thus constructing FPKM-based centroids specific for the ERpHER2n group. The entire ERpHER2n cohort was next reclassified using Spearman correlation in a single sample mode (referred to as PAM50_K0_ subtypes), without any offset, log2 transformation, or gene centering, instead only by correlating each tumor’s FPKM profile to the PAM50_K0_ centroids. As seen in Fig. 6b, the major subtype changes between PAM50_NC_ and PAM50_K0_ were a set of LumA_NC_ tumors shifting to LumB_K0_ and some LumA_NC_ tumors shifting to the Normal_K0_ subtype. In the group of endocrine-treated ERpHER2n tumors, the PAM50_K0_ subtypes were notably associated with different DRFI (Fig. 6c). Moreover, in the same patient group, analysis of biological metagene rank scores showed marked expression patterns for PAM50_K0_ subtypes for the key metagenes representing proliferation, steroid response, and basal gene expression (Fig. 6d). To challenge our hypothesis further, we next selected only the endocrine-treated ERpHER2n LumA_NC_ patients and stratified these by their PAM50_K0_ subtypes. While there was no statistical difference observed for LumA_K0_ versus LumB_K0_ using DRFI as clinical endpoint (Fig. 6e), we observed a marked difference in overall survival between the PAM50_K0_ subtypes in the LumA_NC_ cohort (Fig. 6f). Importantly, in this endocrine-treated ERpHER2n LumA_NC_ group, the PAM50_K0_ subtypes showed an expected biological metagene expression pattern, including slightly elevated proliferation in LumB_K0_ versus LumA_K0_, markedly lower basal expression in LumB_K0_ and higher basal expression in Normal_K0_ versus LumA_K0_ tumors (Fig. 6g). These patterns are consistent with the general trends for PAM50_NC_ subtypes in the full cohort (see, e.g., heatmap in Fig. 3d).

## Discussion

In the current study, we set out to chart the gene expression drivers of PAM50 classification in primary invasive breast cancer. To this end, we applied a gene set centroid perturbation strategy (*leave-oneGeneCluster-out*) to gene expression data from RNA sequencing of 6233 primary breast cancers. Our hypothesis was that the effects of a perturbation to PAM50 classification differed depending on the molecular background. Therefore, we stratified classification effects by underlying molecular clinical subgroups defined by tumor ER, PR, and HER2 status. There are two important methodological strengths of the current study compared to previous reports: (1) the unbiased patient cohort that is representative of population-based disease in South Sweden during 2010–2018, and (2) our rigorous NC classification strategy involving classifying each sample 100 times using 100 different reference sets for normalization/gene centering that are balanced to mimic the original cohort composition of Parker et al.^7^ (see ref. ^14^ for full details).

The typical PAM50 subtype assignment for a tumor is done through the selection of the nearest of five-subtype centroids using a gene expression correlation-based distance metric, i.e., 1-correlation meaning higher correlation equals smaller distance. While there will always be a nearest centroid (with the highest correlation and therefore the assigned subtype), there will also be a second-best subtype for a tumor as distance to each centroid is evaluated. The discrete calling of a PAM50 subtype in a tumor is a pragmatic but simplistic approach as a tumor’s second-best correlation will occasionally be very close to the highest correlation making the subtype call arbitrary in extreme cases as illustrated by Fig. 1g and also noted by Kuilman et al. for the BluePrint molecular subtyping test^24^. As such, tumor subtypes could at times be viewed as the combination of centroid correlations rather than a single nearest centroid much like the ROR score, which is partly calculated by a weighted combination of centroid correlations. This view can be further exemplified by considering the distinctiveness of the best versus second-best subtype, naively conceptualized in our study as the difference in centroid correlation between the two (Fig. 1). Here, it is apparent that the Basal_NC_ subtype is in general the most distinct (largest separation from second-best) subtype across all tested tumor subsets, whereas the Normal_NC_ subtype is typically the least distinct in line with Paquet et al.^16^. For the other PAM50_NC_ subtypes the distinctiveness varies depending on molecular subgroup analyzed. For instance, for HER2E_NC_ the distinctiveness is equivalent to Basal_NC_ in ERnHER2p tumors (i.e., high), lower in ERpHER2p tumors (equivalent to, e.g., LumA_NC_ in this group), while together with Normal_NC_ among the lowest in ERpHER2n tumors. However, although the extent of borderline cases varies between subtypes, all of them contain some cases with insignificant separation between the nearest and second-nearest centroid. As such, the distinctiveness analysis illustrates that a one-class PAM50_NC_ subtype is in many cases a conceptual oversimplification and that tumors instead could be considered as placed somewhere on a continuum between subtypes. Likewise, it illustrates that the support (distinctiveness) for certain subtype calls in typically disparate molecular subgroups can be low (like for HER2E_NC_ in ERpHER2n tumors or LumA_NC_ in TNBC). The latter may then question the relevance of these subtype calls in these molecular subgroups altogether, especially if erroneous sampling or misclassification by conventional pathology markers can be ruled out.

Regarding the pattern of the PAM50_NC_2nd_ subtype in breast cancer, we illustrate that it appears connected to the PAM50_NC_ subtype irrespective of clinical subgroup (Fig. 1). Thus, the PAM50_NC_2nd_ subtype pattern appears as an inherent consequence of how centroids are constructed and inter-correlated (Fig. 1f), which is expected as centroid correlations define the subtypes. For LumA_NC_ and LumB_NC_ tumors the PAM50_NC_2nd_ subtype combinations were also associated with differences in overall survival, but interestingly not in distant recurrence-free interval, in the typical patient target group of current commercial gene expression assays (Fig. 2). As expected, given the nature of the ROR formula (see ref. ^7^), the PAM50_NC_2nd_ subtype in these tumors was associated with differences in ROR-scores, but intriguingly not with obvious differences in tumor size nor patient age that could help explain survival differences. Perhaps more clinically interesting was that within ROR-low and ROR-high the second-best subtype combinations showed trends of different overall survival, but not distant recurrence-free interval (possibly due to follow-up length), for patients. If validated in larger cohorts, this may be useful to further improve clinical risk management as ROR risk groups are a weighted estimate of subtype correlations, proliferation, and tumor size.

To further dissect PAM50_NC_ subtyping we employed a centroid perturbation strategy that excluded sets of co-expressed genes from the PAM50 centroids (*leave-oneGeneCluster-out*). In this strategy, we first identified seven gene clusters, i.e., the gene sets, based on co-expression that showed different expression patterns across subgroups/subtypes and low to moderate correlation to each other (Fig. 3, Supplementary Fig. 1). Gene expression for the three largest gene sets (gene sets 1–3) correlated strongly with proliferation, steroid response, and a basal cell gene expression pattern respectively, as expected given included genes^15^. Identification of PAM50 co-expressed gene sets and the association of these with major biological processes in breast cancer is in line with repeatedly reported mRNA expression patterns of molecular breast cancer phenotypes as already illustrated by Perou et al.^8^. These larger transcriptional themes are naturally reflected in the PAM50 genes in addition to more subtype-specific selected genes (outlined in ref. ^7^). Interestingly, aside from gene sets 1–3, the other gene sets were much smaller (1–2 genes) but still showed distinct gene set scores for certain subtypes consistent with the PAM50 gene selection process. One such example is gene set 7, involving only the *FGFR4* gene, with elevated expression in HER2E_NC_ tumors. *FGFR4* was specifically selected as a gene overexpressed in HER2E_NC_^7^, but it displays a lesser correlation to *ERBB2*, a prototypical HER2E_NC_ gene, in SCAN-B data but also TCGA breast cancers when analyzed through the cBioPortal online tool (Spearman rho = 0.24). In fact, of the genes included in the other gene sets only *ESR1* and *SLC39A6* showed an absolute correlation to *FGFR4* > 0.3 (Spearman rho −0.31 and −0.323, respectively) in the TCGA cohort. Correspondingly, the *GPR160* and *TMEM45B* genes that comprise gene set 6 were selected as genes with reduced expression in Basal tumors^7^. Similar to *FGFR4*/*ERBB2*, the overall correlation between *GPR160* and *TMEM45B* expression in TCGA breast cancers appeared in the lower spectrum (Spearman rho = 0.35). Together, these observations show that the selection of subtype-specific PAM50 genes is not necessarily functionally motivated based on typical co-expression across breast cancers in general. Another observation is that no gene set showed a marked correlation to a stroma, lipid, or immune response associated metagene that is likely more reflective of expression patterns associated with the tumor microenvironment, in line with the original aims of selecting an intrinsic gene list^8^.

The *leave-oneGeneCluster-out* results in the full SCAN-B cohort showed that the Basal_NC_ subtype was resistant to centroid perturbations (Fig. 3e). This repeated observation is consistent with results from the study by Paquet et al. reporting that random perturbations applied not only to NC classification but also to a true PAM50 single sample predictor algorithm resulted in the least number of subtype switches for Basal tumors^16^. Altogether, across TNBC, ERnHER2p, and ERpHER2p tumors it appeared that the most expected (and dominant) PAM50_NC_ subtype (Basal_NC_, HER2E_NC_, and HER2E_NC_, respectively) showed the highest classification stability in the *leave-oneGeneCluster-out* strategy (Fig. 4). In the greater context, this is likely explained by that tumors with these subtypes in their respective clinical subgroup are most representative of the prototypical tumors that once formed the actual centroid values (hence stronger correlations may be expected). Considering this fact, it then becomes crucial to acknowledge from which type of prototypical tumors each centroid was computed when applying and interpreting PAM50 subtyping to all types of breast cancer. In contrast, the same observation was not true for ERpHER2n tumors subtyped as LumA_NC_ or LumB_NC_. The Normal_NC_ subtype presented a special case, as it was the most unstable of all PAM50_NC_ subtypes across clinical subgroups particularly for gene set 3 (basal keratins) suggesting that the expression of these genes is crucial for the subtype. The true nature of the PAM50 Normal_NC_ subtype is debated as it includes both ER-positive and ER-negative tumors and was originally defined by including normal breast tissue samples^7,22^. The Normal_NC_ subtype has been described as being the result of high normal cell content in analyzed bulk tumor specimens or as representing specific histological types of breast cancer, like lobular cancer, or as an additional intrinsic subtype referred to as claudin-low^7,25,26^.

Furthermore, the *leave-oneGeneCluster-out* strategy showed that PAM50_NC_ subtypes had different robustness for the exclusion of different gene sets overall and in specific clinical subgroups. Overall, perturbation of smaller gene sets (gene sets 4–7) caused less tumors to switch, but when it happened the PAM50_perturb_ subtype was typically of the same label as the PAM50_NC_2nd_ (Fig. 4). Detailed analysis of PAM50_perturb_ subtype switching patterns suggests consistency with the intrinsic centroid to centroid correlation and could likely be viewed as a mere illustration of the former (Fig. 1f). For the two *ERBB2*/*HER2*-amplified groups (ERnHER2p and ERpHER2p) the perhaps most interesting observations from the *leave-oneGeneCluster-out* analyses were: (1) the somewhat overall higher stability in ER-negative cases, (2) the general stability of the expected HER2E_NC_ subtype to almost any gene set perturbation, and (3) the lack of direct importance of both the *ERBB2/GRB7* amplicon genes and the *FGFR4* gene in perturbed centroids for classification robustness (Fig. 4 and Supplementary Figs. 3 and 4). Further, while *ERBB2*, *GRB7*, and *FGFR4* are included in the centroids as highly expressed in HER2E^7^, their actual importance in PAM50_NC_ subtyping could be viewed as limited based on our perturbation results. This suggests that the HER2E_NC_ subtype classification in *ERBB2*-amplified disease is likely predominantly dictated by the interplay/interrelationship between other included gene sets, like those capturing proliferation, steroid response, and basal keratin expression, for which HER2E_NC_ tumors often display an intermediate expression pattern compared to Basal_NC_, LumA_NC_, and LumB_NC_ tumors.

In the ERpHER2n group, LumA_NC_ and LumB_NC_ tumors accounted for 90.4% of all tumors. For these tumors, gene sets 1–3 had the greatest impact in the *leave-oneGeneCluster-out* strategy. This finding may be expected considering the well-established role of proliferation as a key divider between LumA_NC_ and LumB_NC_, as well as the importance of ER-signaling for these subtypes^27^. Consistently, the exclusion of gene set 1 (proliferation) caused 49.5% of LumB_NC_ tumors to have a different PAM50_perturb_ subtype that was almost exclusively LumA_perturb_. It might be noted that for these LumB_NC_ tumors, LumA was their PAM50_NC_2nd_ subtype in 69.4% of the cases. A similar pattern was observed when gene set 3 (basal keratins) was excluded in LumB_NC_ tumors. Gene set 3 includes a set of keratin genes (*KRT17*, *KRT5*, *KRT14*) as well as *EGFR*, genes that have repeatedly been shown to be expressed in basal-like tumor cells by in situ analyses^28^, but also in the Normal_NC_ subtype by mRNA profiling (e.g., ref. ^25^). As seen in Fig. 3b, gene set 3 has the highest average PAM50 centroid values for the Normal and Basal centroids, while intermediate for LumA and lower for LumB in line with previous reports^25^. Thus, while luminal tumor cells are likely not expressing these specific keratin markers, they will still have an impact on PAM50 classification due to the reliance on classification on relative mRNA expression through the process of gene centering, particularly for LumB_NC_. In comparison, in recent rule-based PAM50 classifiers (based on intrinsic gene pairs and not relative mRNA expression) reported by us, *KRT17*, *KRT5*, *KRT14*, and *EGFR* were selected in gene rules for the LumB and Normal (*KRT5* and *KRT14*) subtypes, but not at all for LumA^14^.

While the *leave-oneGeneCluster-out* strategy can inform about which gene sets appear important for classification and which tumors appear stable to any perturbation, an obvious question is whether this has any clinical impact or can be used to refine classification in the future. To address the first question, we performed survival analysis for each gene set in each clinical group comparing patients whose tumors switched to those that did not. This analysis showed that gene sets 1–3 switches had prognostic value in TNBC and ERpHER2n patients but not in any of the HER2-positive groups (Fig. 5a–e). In this context, implementing the *leave-oneGeneCluster-out* strategy as an add-on to PAM50_NC_ classification is a straightforward computational exercise that could label a PAM50_NC_ tumor as “stable/prototypical”. To address whether the *leave-oneGeneCluster-out* could be used to refine PAM50 classification we tested the hypothesis of creating new ERpHER2n specific PAM50 centroids based on perturbation stable tumors, for which classification would subsequently rely only on Spearman correlation without gene centering, a step demonstrated to be a frail trait of centroid prediction^16,20^. This exercise demonstrated that this could be possible and that the inferred new PAM50 subtypes (PAM50_K0_) could refine the existing LumA_NC_ subtype in ERpHER2n tumors with respect to both gene expression patterns but also patient overall survival after endocrine therapy (Fig. 6).

A limitation of the current study lies in the gene set size of the PAM50 centroids. Intuitively, removing a large proportion of genes from the centroids will inevitably increase subtype switching as shown by Paquet et al.^16^. This limits the interpretation potential of excluding combinations of gene sets (e.g., gene sets 1 and 2, representing 60% of the gene content) as correlations could drop below meaningful levels. Moreover, the interpretation of correlations to perturbed centroids should also be done with caution as omitting genes certainly changes subtype centroids to something other than what they originally are. However, here it is worth noting that centroid correlations for tumors that either had the same PAM50_NC_ and PAM50_perturb_ subtype or different when excluding a particular gene set did not consistently decrease or increase in the clinical subgroups (Supplementary Figs. 6–9). Instead, a highly variable pattern of increased and decreased correlations between the PAM50_NC_ and the PAM50_perturb_ subtype after reclassification was observed. Examples are even seen where tumors with the same PAM50_NC_ and PAM50_perturb_ subtype show a higher correlation to the reduced PAM50_perturb_ centroid. Moreover, we note that while not always the case, when a tumor had a different PAM50_perturb_ subtype in the *leave-oneGeneCluster-out* strategy, it was often the same subtype as the corresponding PAM50_NC_2nd_ (Fig. 4d, Supplementary Figs. 2–5). Together these observations support that the taken perturbation approaches can bring meaningful insights. A final limitation of mRNA-based bulk tissue analysis, which we cannot properly address, lies in the sampling procedure and the tissue heterogeneity and cellularity. This has repeatedly been reported to affect PAM50 subtyping in both frozen tissue (see ref. ^26^), but also for the Prosigna assay itself which is based on macro-dissected tumor tissue^29^.

In summary, in the current study, we have analyzed features of PAM50 subtype classification in the context of molecular-clinical subgroups. This task is complicated by the tight interrelationships between gene/gene sets in the original PAM50 centroids that define classification when applied to tumors using relative expression and correlation. As illustrated in Fig. 1g, for many tumors the discrete subtype assignment of LumA_NC_ or LumB_NC_ is clearly not represented by distinct proximity to a sole single centroid. Hence, a tumor could be viewed as either one of the subtypes or perhaps better as a combination of all subtypes. On the other hand, many of the results presented in this study appear as logical illustrations of the framework set by the centroids and the usage of correlation as a similarity metric. Moreover, an innate discrete subtype of a tumor may also be challenged considering the continuum of expression patterns governing subtype calls (like expression of proliferation-related genes). In this study, we show that perturbations to the PAM50_NC_ classification have a different impact depending on the underlying ER, PR, and HER2 status of the tumor and the excluded gene set. Moreover, we show that the PAM50_perturb_ pattern is different between PAM50_NC_ subtypes within a clinical subgroup, but also within the PAM50_NC_ subtypes themselves depending on the excluded gene set, and that this can have prognostic associations. The reasons for this are likely several, including the underlying biological processes in breast cancer that are at least partly reflected in the PAM50 gene set, but also presumably the specific selection of certain PAM50 genes to represent particular subtypes, and that each centroid was created from a set of prototypical tumors (e.g., for the Basal subtype these were mainly TNBC tumors). Interestingly, the smaller gene sets typically do not correlate with major transcriptional programs in breast cancer and are not apparently biologically co-expressed either. Deconstructing their importance and relevance in subtyping remains a topic for further investigation, as they clearly are of importance to classification in certain situations and are potentially more vulnerable to technical failures due to a lack of redundant co-expressed genes.

An improved understanding of the commonly used PAM50 subtyping scheme in breast cancer and systematic illustrations of correlation interplay such as those presented here will aid the interpretation of tumors with seemingly disparate classifications, like clinically defined ERpHER2n tumors that are subtyped as PAM50 Basal_NC_, and whether these represent true biological entities. Moreover, the trends we observed in overall survival in ROR groups for the combination of PAM50_NC_ and PAM50_NC_2nd_ subtypes, as well as the demonstrated prognostic differences based on gene set perturbation, and the demonstrated potential to refine PAM50 subtyping based on tumors stable to gene set perturbations may be of interest for future clinical management. Importantly, studies such as this one challenge the conception that PAM50 subtypes are individual discrete classes and call for a shift in the way we approach the results of this classification.

## Methods

### Unselected population-based breast cancer cohort

A total of 6233 patients diagnosed with primary invasive breast tumors and enrolled in the Sweden Cancerome Analysis Network–Breast (SCAN-B) study^30,31^ (ClinicalTrials.gov ID NCT02306096) from 2010 to 2018 with curated RNA sequencing data and complete clinicopathological and PAM50 data (specifically PAM50 classification as Basal, HER2E, LumA, LumB, or Normal, ER, PR, HER2, and nodal status, treatment indication, and patient follow-up) available in Staaf et al.^14^ were included. The included cohort is hereafter referred to as SCAN-B. The 6233 patients comprise 93.6% of the 6660-sample early-stage follow-up cohort (one patient – one tumor RNA sequencing profile) defined in ref. ^14^ from the total set of 9206 RNA sequencing profiles in ref. ^14^. Clinicopathological and molecular characteristics for the 6233 patients’ tumors are detailed in Supplementary Table 1. Specific patient inclusion and exclusion criteria for the SCAN-B cohort are reported in the original publication^14^. Patients in this cohort have previously been shown to be representative of the underlying breast cancer population of the healthcare region in which they were enrolled^14,22^. The PAM50 classification used in this study is based on the five-subtype system (Basal, HER2E, LumA, LumB, Normal) using the NC classification methodology reported in ref. ^14^ (therein termed NCN). In the classification approach described in ref. ^14^, each tumor is subtyped 100 times using 100 different reference sets for centering, resulting in 100 correlations to each PAM50 centroid from which a majority subtype vote is determined. The majority subtype is hereon referred to as a tumor’s PAM50_NC_ subtype (Basal_NC_, HER2E_NC_, LumA_NC_, LumB_NC_, Normal_NC_). For the calculation of a tumor’s correlation to the best (PAM50_NC_) and second-best (PAM50_NC_2nd_) NC subtype, the average correlation per centroid of the 100 correlation values was used. This average value was also used to determine the PAM50_NC_2nd_ subtype for a tumor. Patients were divided into four clinically relevant subgroups (with different therapy options) according to ER, PR, and HER2 status (p = positive, n = negative) available from the clinical cancer registry: (1) TNBC, (2) ERnHER2p, (3) ERpHER2p, and (4) ERpHERn.

### Ethical approval

All SCAN-B enrolled patients provided written informed consent prior to study inclusion as described in Staaf et al.^14^. Ethical approval was given for the SCAN-B study (approval numbers 2009/658, 2010/383, 2012/58, 2013/459, 2015/277) by the Regional Ethical Review Board in Lund, Sweden, governed by the Swedish Ethical Review Authority, Box 2110, 750 02 Uppsala, Sweden.

### PAM50 gene set clustering

To identify co-expressed gene clusters among the PAM50 genes we used SRIQ clustering^32^ of FPKM data from all RNA sequencing profiles reported in ref. ^14^ (*n* = 9206). SRIQ is an unsupervised clustering method that incorporates concepts from random forest machine learning as well as quality threshold- and k-nearest neighbor clustering to identify a core cluster of samples or genes that share common patterns without requiring prior knowledge of the data or a predefined number of clusters. The rationale behind using the larger set of 9206 RNA sequencing profiles was to have as many breast cancer expression profiles as possible for the gene clustering, acknowledging that replicates exist among the 9206 profiles as described in ref. ^14^. SRIQ analysis identified six core gene clusters comprising 45 of 50 PAM50 genes (see ref. ^32^ for details about core clustering). The other five genes, *MYC*, *MMP11*, *BAG1*, *MDM2*, and *BLVRA*, were not included in any SRIQ core cluster. One of the six SRIQ clusters comprised *ERBB2* (17q12), *GRB7* (17q12), and *FGFR4* (5q35.2) and was manually split into two clusters (*ERBB2*/*GRB7* and *FGFR4*, respectively). The decision to split this particular SRIQ cluster was based on: (1) the genomic proximity of *ERBB2* and *GRB7* in the same minimally amplified region (17q12, see ref. ^33^) versus *FGFR4*, (2) the key role of the *ERBB2* locus for clinical management of breast cancer, (3) the specific addition of *FGFR4* as a HER2E prototype gene to the original PAM50 centroids^7^, and (4) the expression correlation of the three genes where *ERBB2*/*GRB7* are tightly correlated (Pearson correlation of 0.9 across 9206 RNA sequencing profiles) whereas *FGFR4*/*ERBB2* showed only a Pearson correlation of 0.285 across the 9206 assays. This split resulted in seven final gene clusters to be evaluated. Gene set scores for each tumor were calculated as the average log2 (FPKM+0.1 offset) value of genes included in the respective gene cluster (i.e., no gene centering). Functional analysis of the gene clusters was performed by: (1) pathway enrichment analysis using Enrichr (v3.1)^34,35^ accessing the KEGG pathway^36,37^ and Gene Ontology Consortium databases^38,39^ with an adjusted *p*-value cut-off of *p* < 0.05, and (2) correlation across samples of gene set scores and rank scores for eight biological metagenes reported by Fredlund et al.^15^ calculated as defined by Nacer et al.^40^.

### PAM50 reclassification following a leave-oneGeneCluster-out strategy

PAM50 reclassification was performed by modifying the method outlined in ref. ^14^ following a *leave-oneGeneCluster-out* strategy where individual SRIQ-derived gene sets were sequentially excluded. Three different gene-matched data matrices were used: (1) the PAM50 centroids from Parker et al.^7^, (2) a matrix including 100 defined reference sets for gene centering as defined in ref. ^14^, and (3) a matrix for the samples to be classified. The strategy was based on excluding one of the SRIQ-derived PAM50 gene clusters at a time from the centroid, reference set, and expression matrices, creating reduced gene matrices. For each sample NC classification using the remaining genes was performed 100 times using the 100 defined reference sets for gene centering as defined in ref. ^14^. The genes specifically included in the seven SRIQ core gene sets formed the background centroid gene content for these analyses. The resulting subtype, PAM50_perturb_, was called Basal_perturb_, HER2E_perturb_, LumA_perturb_, LumB_perturb_, and Normal_perturb_. A sample was called as having a subtype switch if the PAM50_NC_ subtype was observed in ≤50% of the 100 PAM50_perturb_ classifications and we used no minimum correlation cut-off for subtyping.

### cBioPortal analyses

For gene-gene comparisons in TCGA breast cancers, we used the cBioPortal online tool (www.cbioportal.org). For correlation analyses, the option of log-transformed mRNA expression z-scores compared to the expression distribution of all samples (RNA Seq V2 RSEM) was used for 1082 tumors.

### Statistical methods

All *p*-values reported are two-sided and were compared to a level of significance of 0.05 unless otherwise specified. Boxplot elements correspond to: (1) center line = median, (2) box limits = upper and lower quartiles, (3) whiskers = 1.5x interquartile range. Correlations were computed using Spearman correlation unless otherwise specified.

### Survival analysis

Survival analyses were performed in R (v4.2.2) using the *survival* (v3.4.0) and *survminer* (v0.4.9) packages with overall survival (OS) and distant recurrence-free interval (DRFI) as primary endpoints obtained from ref. ^14^. Survival curves were estimated using the Kaplan–Meier method and compared using the log-rank test. Cox proportional hazard ratios were computed using the *coxph* function in R.

### Reporting summary

Further information on research design is available in the Nature Research Reporting Summary linked to this article.

### Supplementary information

SupplementaryInformation

Supplementary Table 1

Supplementary Table 2

Reporting Summary

## Data Availability

Clinical, molecular, and processed RNA sequencing data (fragments per kilobase million, FPKM) were obtained from an open-access repository associated with the study by Staaf et al.^14^.

## Code availability

All analyses were performed using open-source software such as Java and the R statistical language. Specific sources of code used in this study for the SRIQ co-expressed gene clusters analysis can be found at https://github.com/StaafLab/SRIQ.
