# Supplementary material for: Perturbation and stability of PAM50 subtyping in population-based primary invasive breast cancer
Source: NPJ Breast Cancer. 2023 Oct 19;9:83. doi: 10.1038/s41523-023-00589-0 (PMC10587090; doi:10.1038/s41523-023-00589-0)
Supplement: Supplementary file 1 — SupplementaryInformation [file 41523_2023_589_MOESM1_ESM.pdf]

A) TNBC

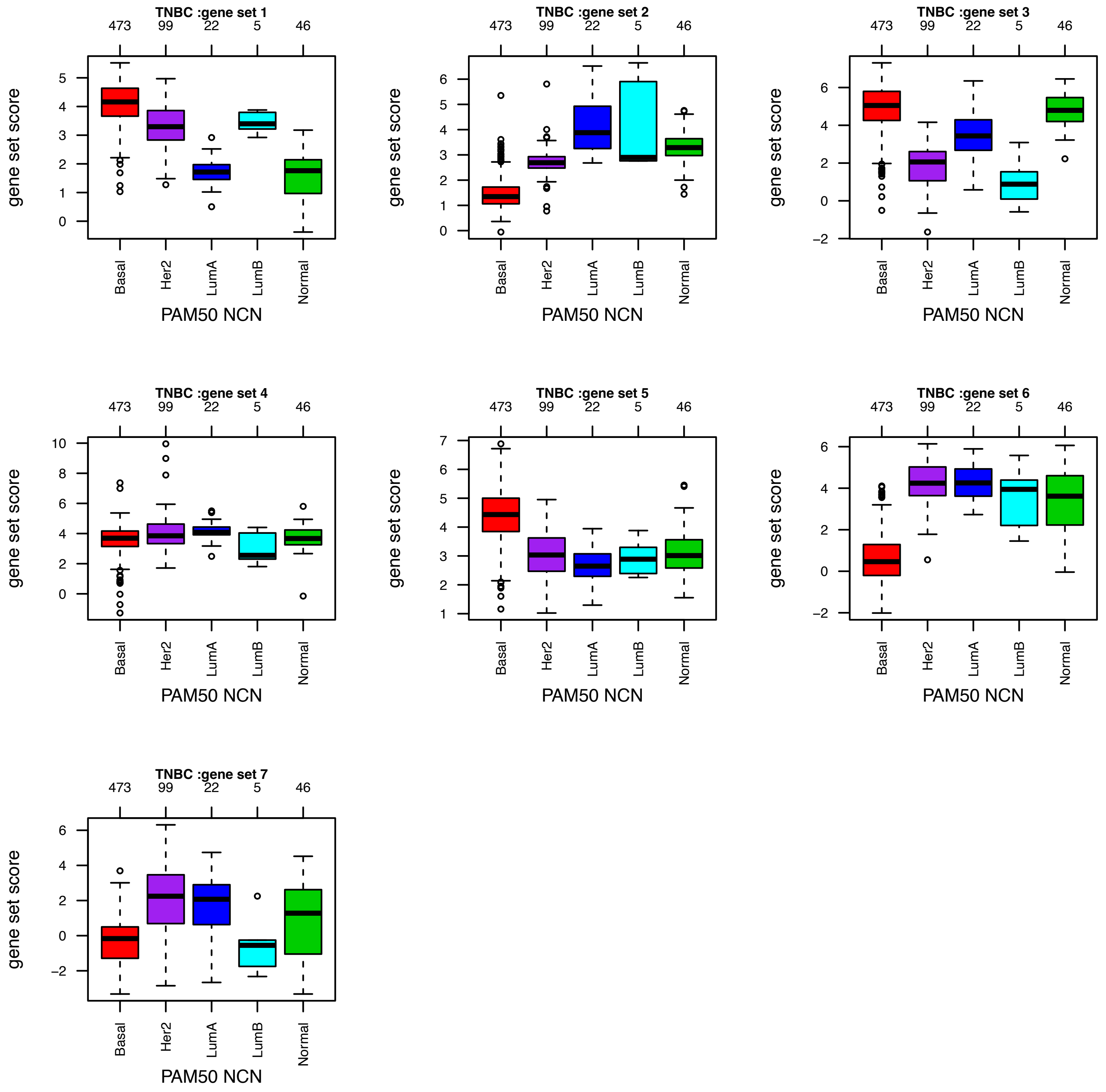

Supplementary Figure 1. Gene set score expression in breast cancer subgroups defined by ER, PR, and HER2 status and stratified by PAM50 subtypes, and versus biological expression metagenes. The gene set score is the average log2 FPKM of genes included in the gene set. (**A**) TNBC tumors. (**B**) ERnHER2p tumors (**C**) ERpHER2p tumors. (**D**) ERpHER2n tumors. (**E**) Mean gene set expression (FPKM) of gene set 1, 2, and 3 versus rank scores for specific biological metagenes representative of proliferation, steroid response, and basal/keratin expression as defined by Fredlund et al. BCR 2012. Correlations calculated using Spearman correlation using all 6233 tumors.
Boxplot elements correspond to: (i) center line = median, (ii) box limits = upper and lower quartiles, (iii) whiskers = 1.5x interquartile range.

B) ERnHER2p

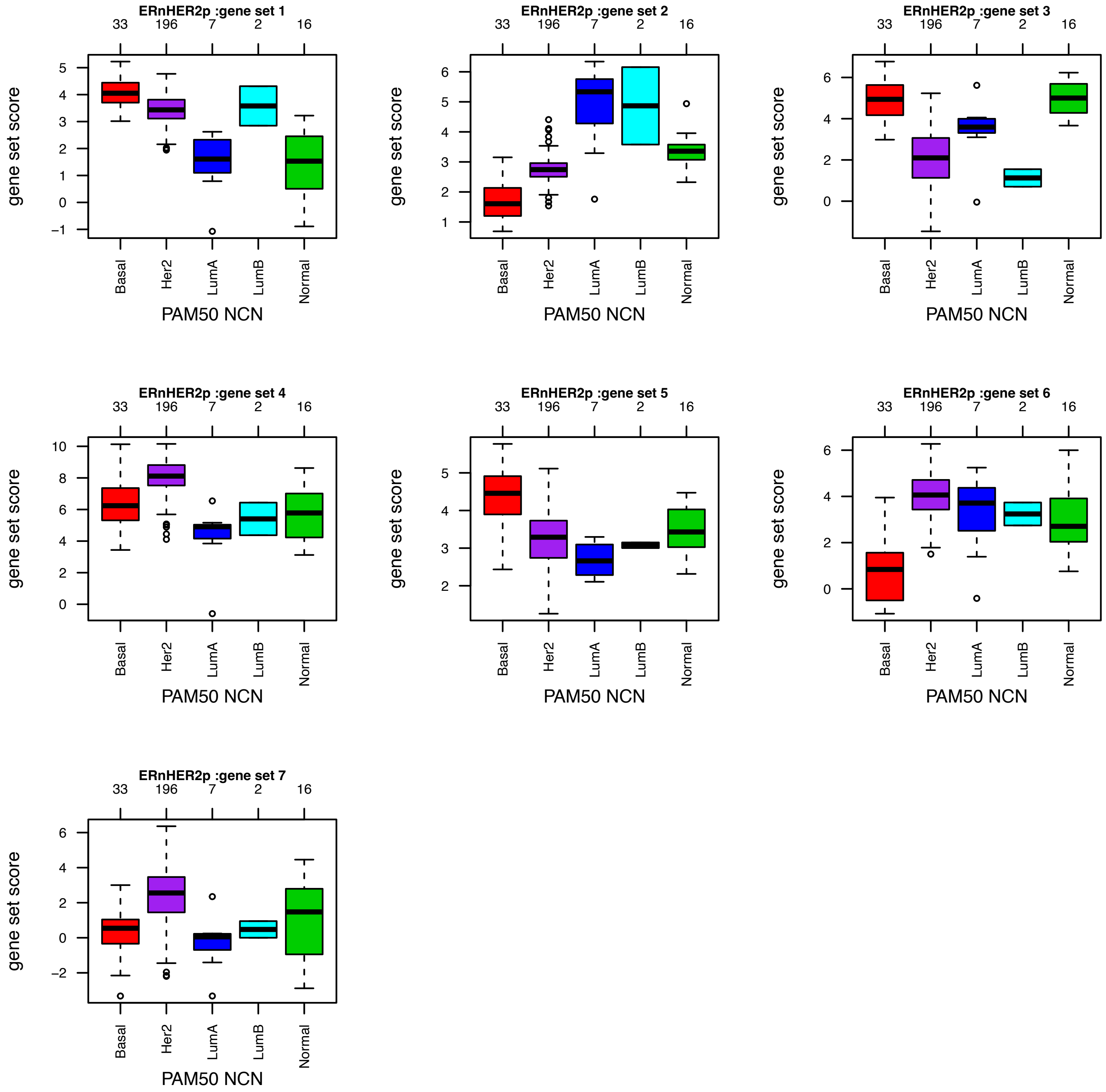
ERnHER2p :gene set 1
ERnHER2p :gene set 2
ERnHER2p :gene set 3
ERnHER2p :gene set 4
ERnHER2p :gene set 5
ERnHER2p :gene set 6
ERnHER2p :gene set 7
33
196
7
2
16
gene set score
Basal
Her2
LumA
LumB
Normal
PAM50 NCN

C) ERpHER2p

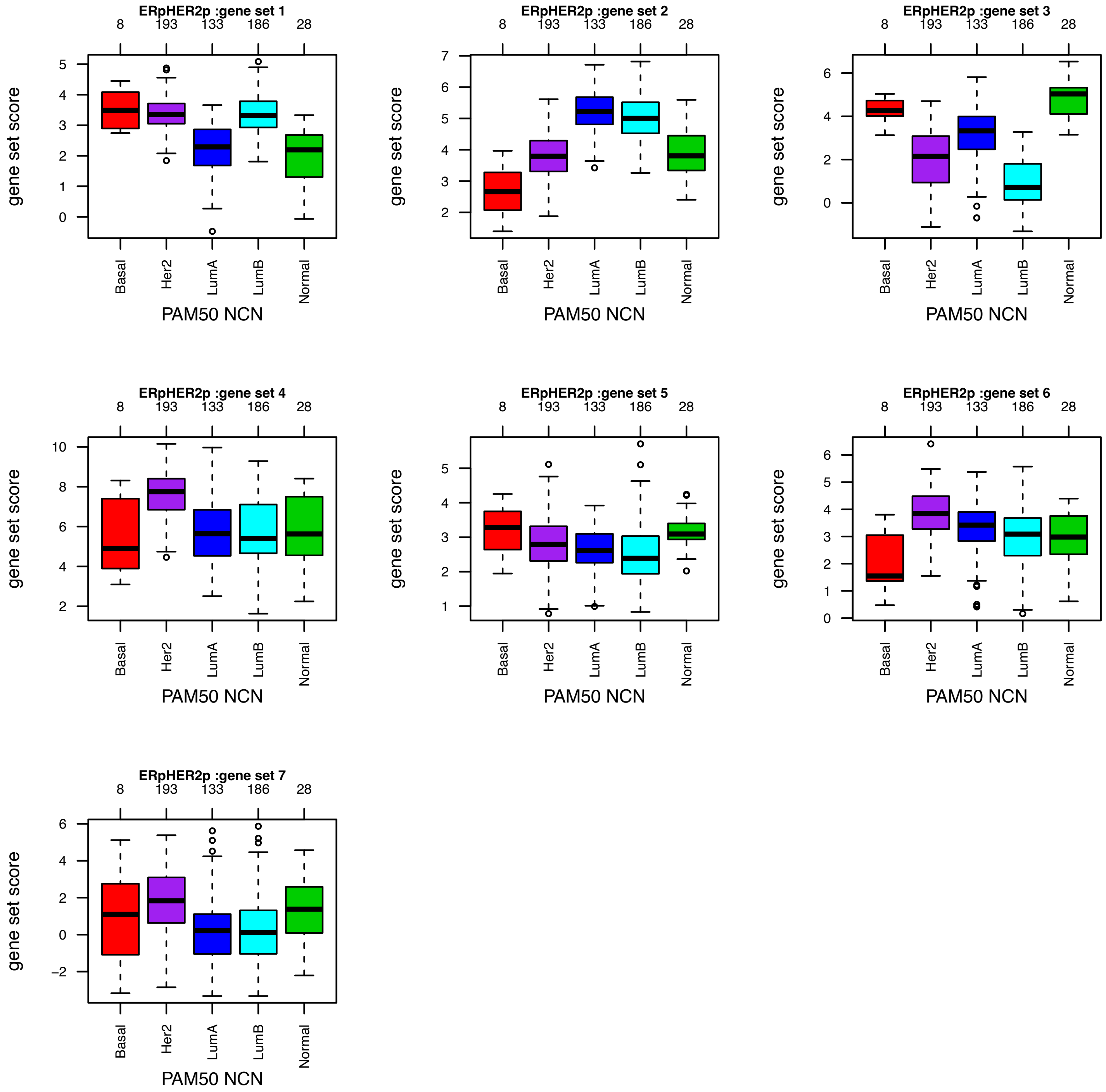

ERpHER2p :gene set 1
8
193
133
186
28
gene set score
0
1
2
3
4
5
Basal
Her2
LumA
LumB
Normal
PAM50 NCN
ERpHER2p :gene set 2
8
193
133
186
28
gene set score
2
3
4
5
6
7
Basal
Her2
LumA
LumB
Normal
PAM50 NCN
ERpHER2p :gene set 3
8
193
133
186
28
gene set score
0
2
4
6
Basal
Her2
LumA
LumB
Normal
PAM50 NCN
ERpHER2p :gene set 4
8
193
133
186
28
gene set score
2
4
6
8
10
Basal
Her2
LumA
LumB
Normal
PAM50 NCN
ERpHER2p :gene set 5
8
193
133
186
28
gene set score
1
2
3
4
5
Basal
Her2
LumA
LumB
Normal
PAM50 NCN
ERpHER2p :gene set 6
8
193
133
186
28
gene set score
0
1
2
3
4
5
6
Basal
Her2
LumA
LumB
Normal
PAM50 NCN
ERpHER2p :gene set 7
8
193
133
186
28
gene set score
-2
0
2
4
6
Basal
Her2
LumA
LumB
Normal
PAM50 NCN

D) ERpHER2n

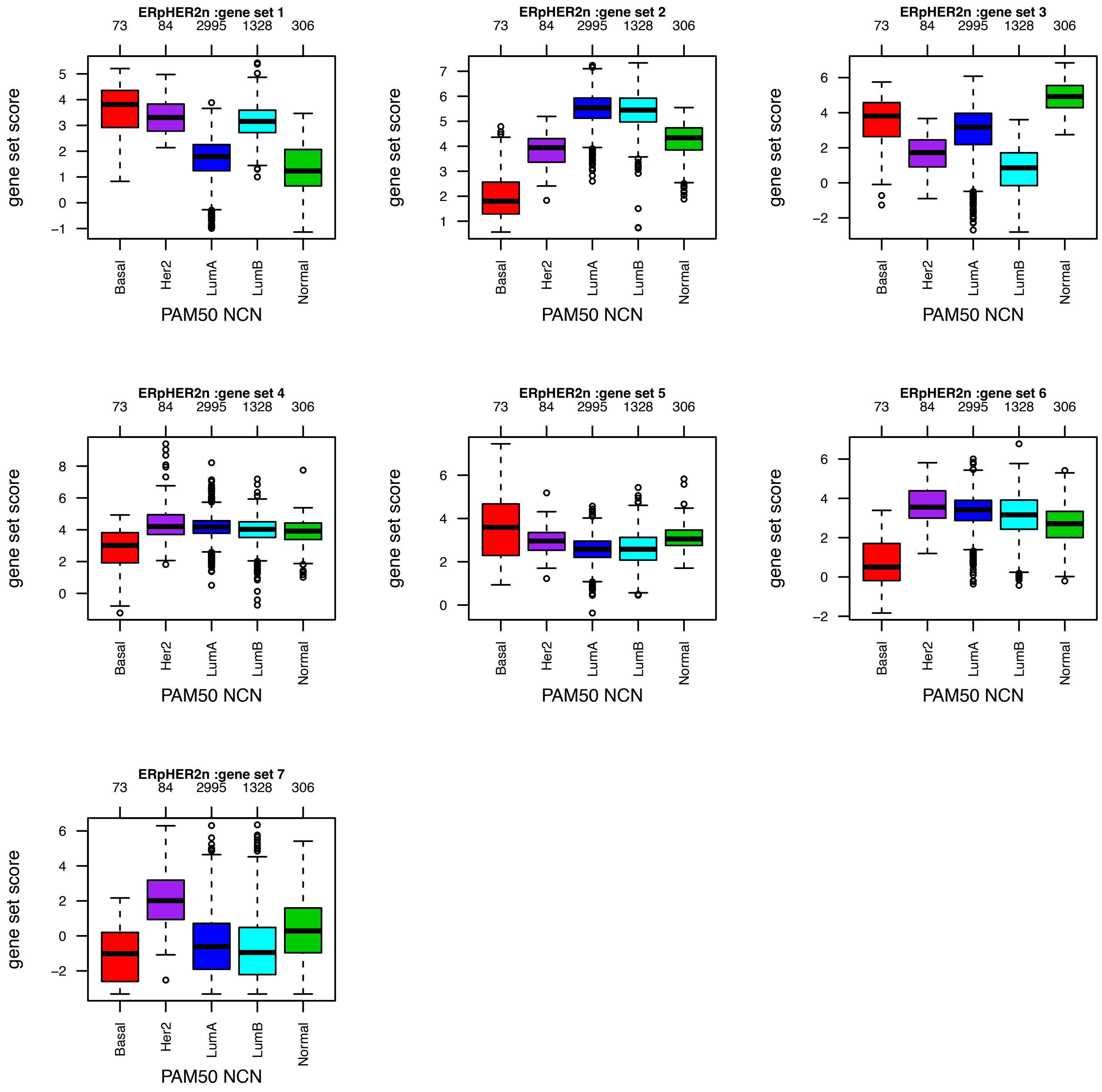

ERpHER2n :gene set 1
ERpHER2n :gene set 2
ERpHER2n :gene set 3
ERpHER2n :gene set 4
ERpHER2n :gene set 5
ERpHER2n :gene set 6
ERpHER2n :gene set 7
73 84 2995 1328 306
gene set score
Basal
Her2
LumA
LumB
Normal
PAM50 NCN

E) Gene set scores vs Fredlund metagenes

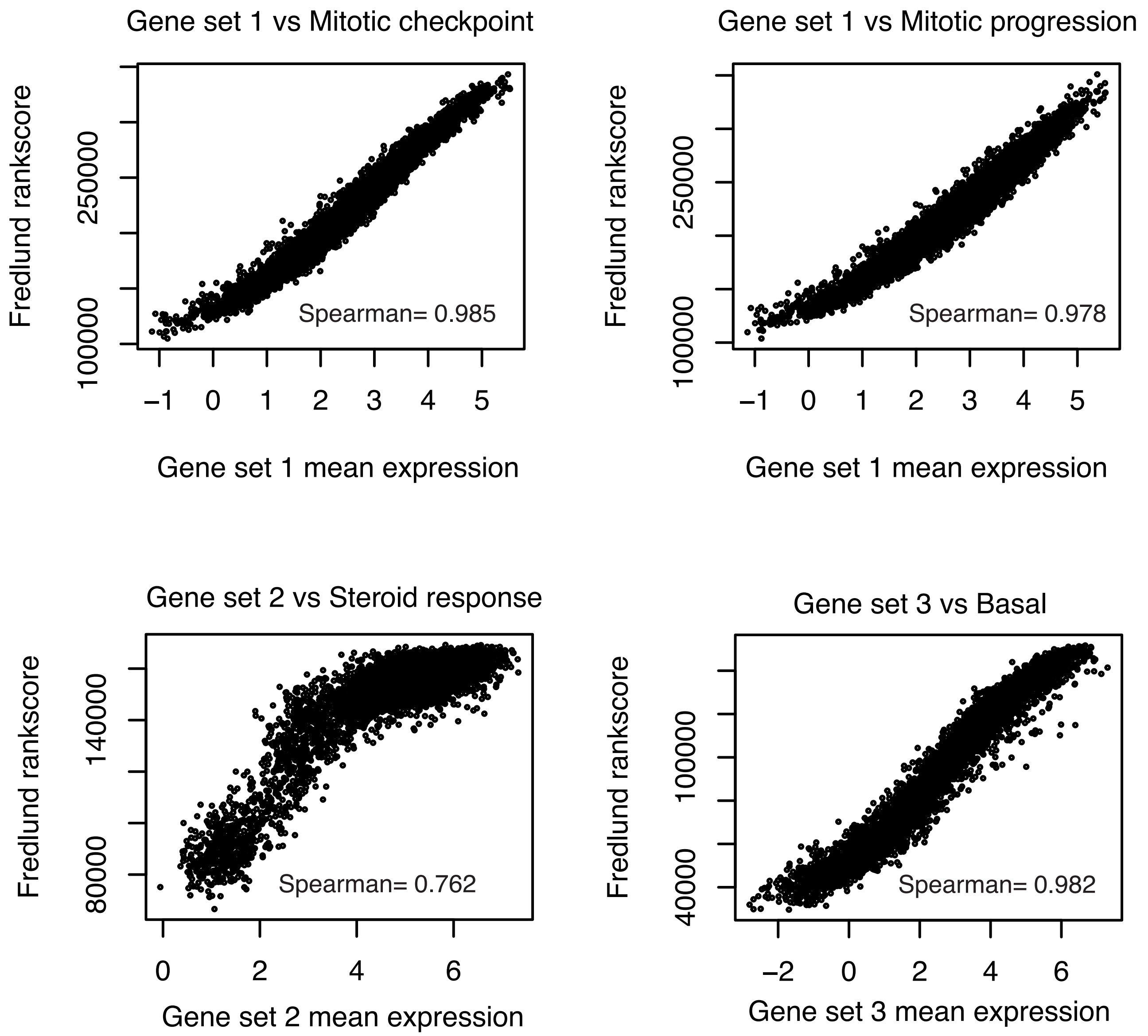

Supplementary Figure 2. TNBC

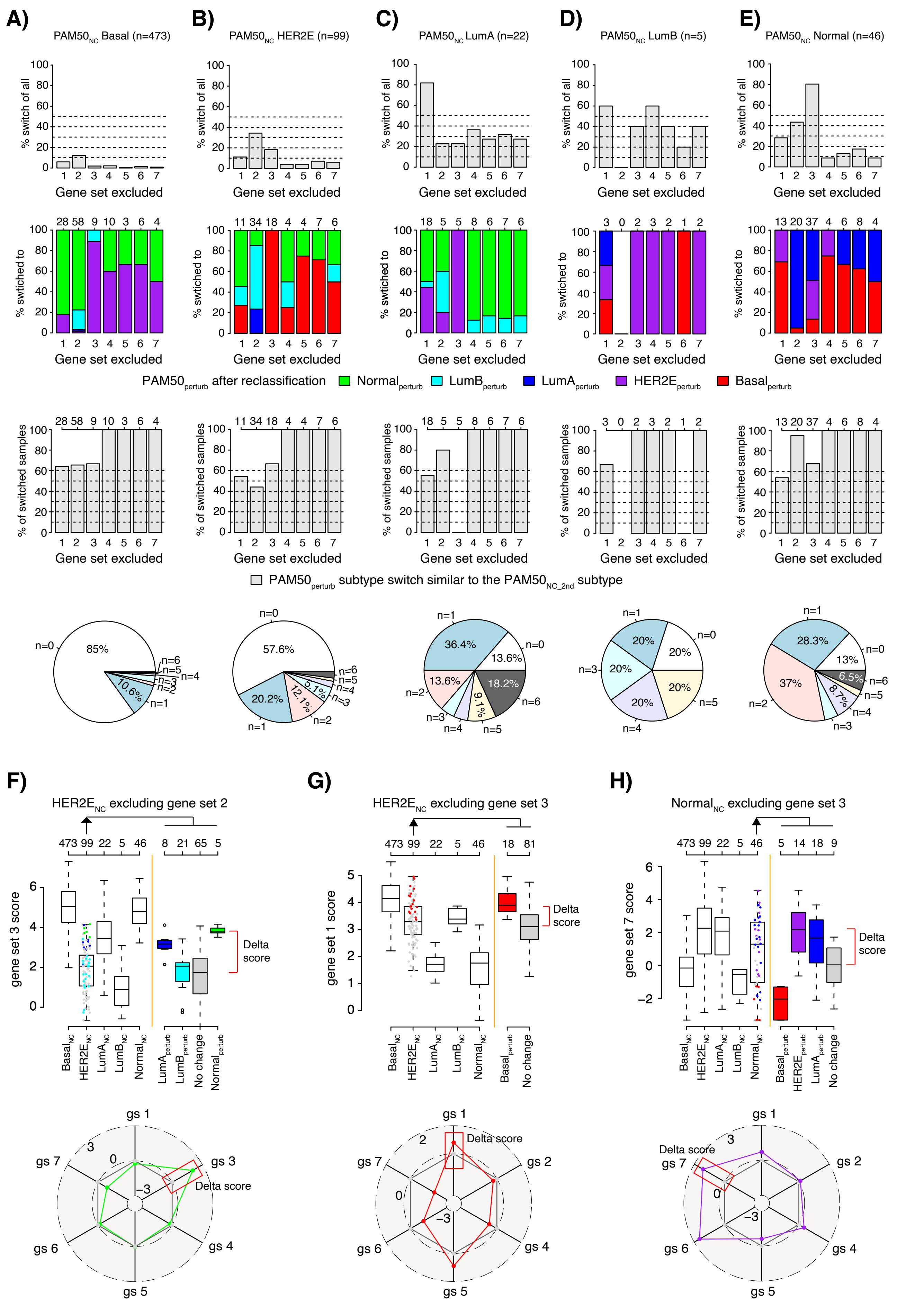

Supplementary Figure 2. $PAM50_{perturb}$ subtype switch effect of the leave-oneGeneCluster-out strategy in TNBC. A-E) Top panels show the proportion of TNBC tumors with a $PAM50_{perturb}$ subtype different from $PAM50_{NC}$, i.e., switching subtype, when excluding a specific gene set in *leave-oneGeneCluster-out reclassification*. Second level panels show the distribution of the $PAM50_{perturb}$ subtypes in tumors that switched in the top panel, number at top represents total number of samples that switched subtype. Third level panels show how many of the tumors that switched subtype after gene set exclusion had a $PAM50_{perturb}$ subtype similar to the $PAM50_{NC\_2nd}$ subtype, number at top represents total number of samples that switched subtype. Bottom pie charts show the proportion of samples that switched n number of times across all gene set exclusions, e.g., size of n=6 equals the proportion of all tumors that had a $PAM50_{perturb}$ switch in 6 out of 7 gene set exclusions. $PAM50_{perturb}$ subtype switch was calculated in **A**) 473 $Basal_{NC}$ tumors, **B**) 99 $HER2E_{NC}$ tumors, **C**) 22 $LumA_{NC}$ tumors, **D**) five $LumB_{NC}$ tumors, and **E**) 46 $Normal_{NC}$ tumors. **F**) Top panel: boxplots of gene set 3 scores for all TNBCs stratified by $PAM50_{NC}$ subtypes (left part, representing original data), and for $HER2E_{NC}$ TNBCs stratified by the $PAM50_{perturb}$ subtype after exclusion of gene set 2 (right part, representing perturbed subtypes). The difference in median gene set 3 score for perturbed cases with a $HER2E_{perturb}$ subtype (No change, gray) versus tumors with a $Normal_{perturb}$ subtype is illustrated. Bottom panel: Spider chart of all median gene set (gs 1-7) expression scores for $HER2E_{NC}$ tumors with a $Normal_{perturb}$ subtype (n=5) after exclusion of gene set 2 (gs2 excluded from the chart). Gene set scores are centered to the group of tumors not shifting (gray in top panel boxplots, representing the inner ring with value 0). Thus, rings correspond to delta score values for a specific gene set (axes). Axes in the spider chart portray the median delta score difference for each gs for $Normal_{perturb}$ vs $HER2E_{perturb}$ tumors after gene set 2 exclusion. The gs 3 delta score difference highlighted in the top boxplot is highlighted in the corresponding axis of the spider chart for the $Normal_{perturb}$ tumors (red box). **G**) Similar illustrations as in F for $HER2E_{NC}$ tumors after exclusion of gene set 3. Boxplots show scores for gs 1, with the gs 1 delta score difference between $Basal_{perturb}$ (n=18) and $HER2E_{perturb}$ (No change) highlighted also in the spider chart for tumors with a $Basal_{perturb}$ subtype (n=18). **H**) Similar illustration as in F for $Normal_{NC}$ tumors after exclusion of gene set 3. Boxplots show scores for gs 7, with the delta score difference between $HER2E_{perturb}$ (n=14) and $Normal_{perturb}$ (No change) highlighted also in the spider chart for tumors with a $HER2E_{perturb}$ subtype.

Boxplot elements correspond to: (i) center line = median, (ii) box limits = upper and lower quartiles, (iii) whiskers = 1.5x interquartile range.

Supplementary Figure 3. ERnHER2p

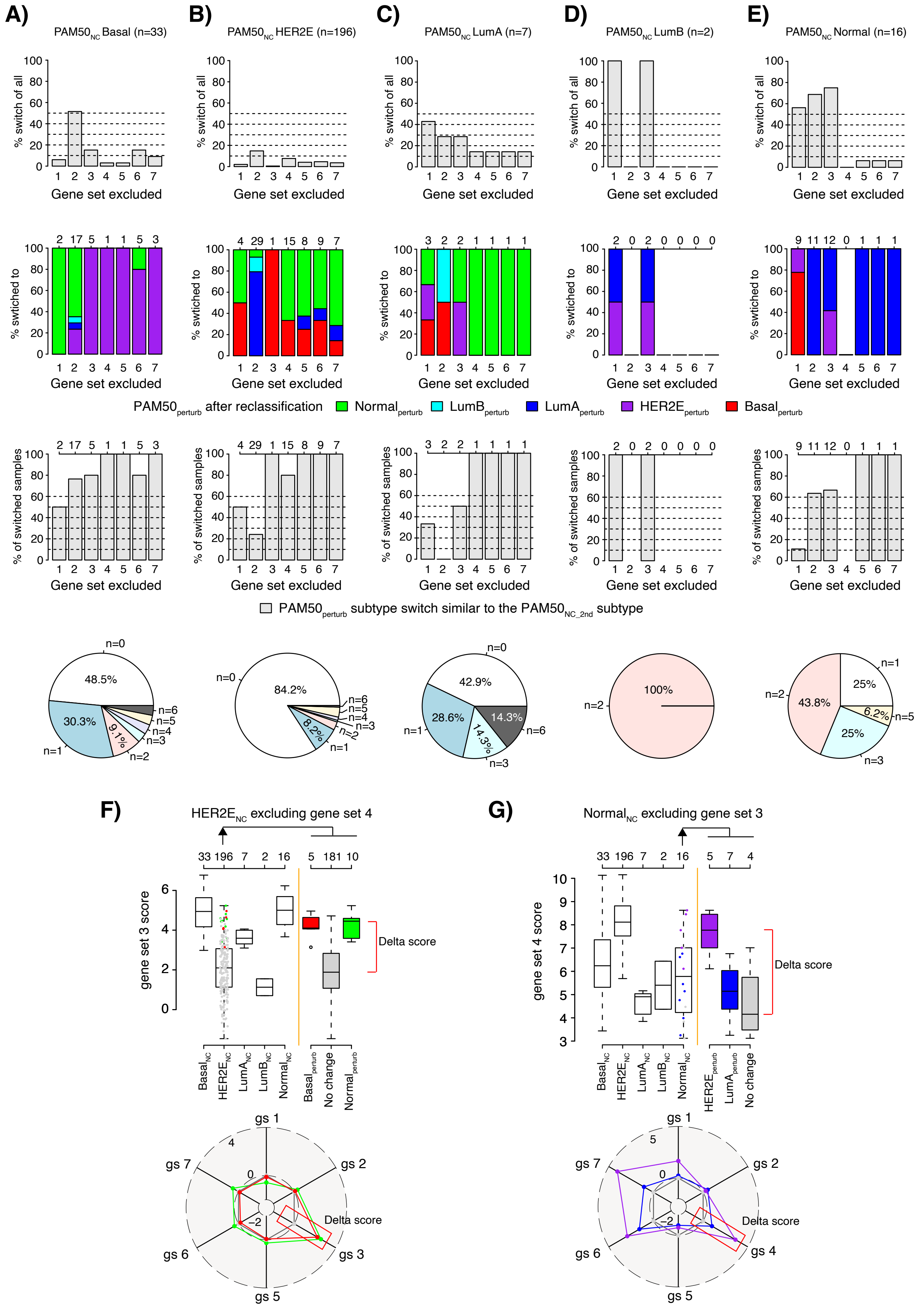

Supplementary Figure 3. PAM50perturb subtype switch effect of the leave-oneGeneCluster-out strategy in ERnHER2p disease. **A-E**) Top panels show the proportion of ERnHER2p tumors with a $PAM50_{perturb}$ subtype different from $PAM50_{NC}$, i.e., switching subtype, when excluding a specific gene set in *leave-oneGeneCluster-out* reclassification. Second level panels show the distribution of the $PAM50_{perturb}$ subtypes in tumors that switched in the top panel, number at top represents total number of samples that switched subtype. Third level panels show how many of the tumors that switched subtype after gene set exclusion had a $PAM50_{perturb}$ subtype similar to the $PAM50_{NC\_2nd}$ subtype, number at top represents total number of samples that switched subtype. Bottom pie charts show the proportion of samples that switched n number of times across all gene set exclusions, e.g., size of n=6 equals the proportion of all tumors that had a $PAM50_{perturb}$ switch in 6 out of 7 gene set exclusions. $PAM50_{perturb}$ subtype switch was calculated in **A**) 33 $Basal_{NC}$ tumors, **B**) 196 $HER2E_{NC}$ tumors, **C**) seven $LumA_{NC}$ tumors, **D**) two $LumB_{NC}$ tumors, and **E**) 16 $Normal_{NC}$ tumors. **F**) Top panel: boxplots of gene set 3 scores for all ERnHER2p tumors stratified by $PAM50_{NC}$ subtypes (left part, representing original data), and for $HER2E_{NC}$ tumors stratified by the $PAM50_{perturb}$ subtype after exclusion of gene set 4 (right part, representing perturbed subtypes). The difference in median gene set 3 score for perturbed cases with a $HER2E_{perturb}$ subtype (No change, gray) versus tumors with a $Normal_{perturb}$ or $Basal_{perturb}$ subtype is illustrated. Bottom panel: spider chart of all median gene set (gs 1-7) expression scores for $HER2E_{NC}$ tumors with a $Normal_{perturb}$ (n=10) or $Basal_{perturb}$ (n=5) subtype after exclusion of gene set 4 (gs4 excluded from the chart). Rings correspond to delta score values for a specific gene set centered to the no change tumors (ring value 0). The gs 3 delta score difference highlighted in the top boxplot is highlighted in the corresponding axis of the spider chart for the $Normal_{perturb}$ and $Basal_{perturb}$ tumors (red box). **G**) Similar illustrations as in F for $Normal_{NC}$ ERnHER2p tumors after exclusion of gene set 3. Boxplots show gs 4 (*ERBB2*/*GRB7*) scores, with the gs 4 delta score difference between $HER2E_{perturb}$ (n=5) and $Normal_{perturb}$ (No change) highlighted also in the corresponding spider chart showing all gene sets except gs3.
Boxplot elements correspond to: (i) center line = median, (ii) box limits = upper and lower quartiles, (iii) whiskers = 1.5x interquartile range.

Supplementary Figure 4. ERpHER2p

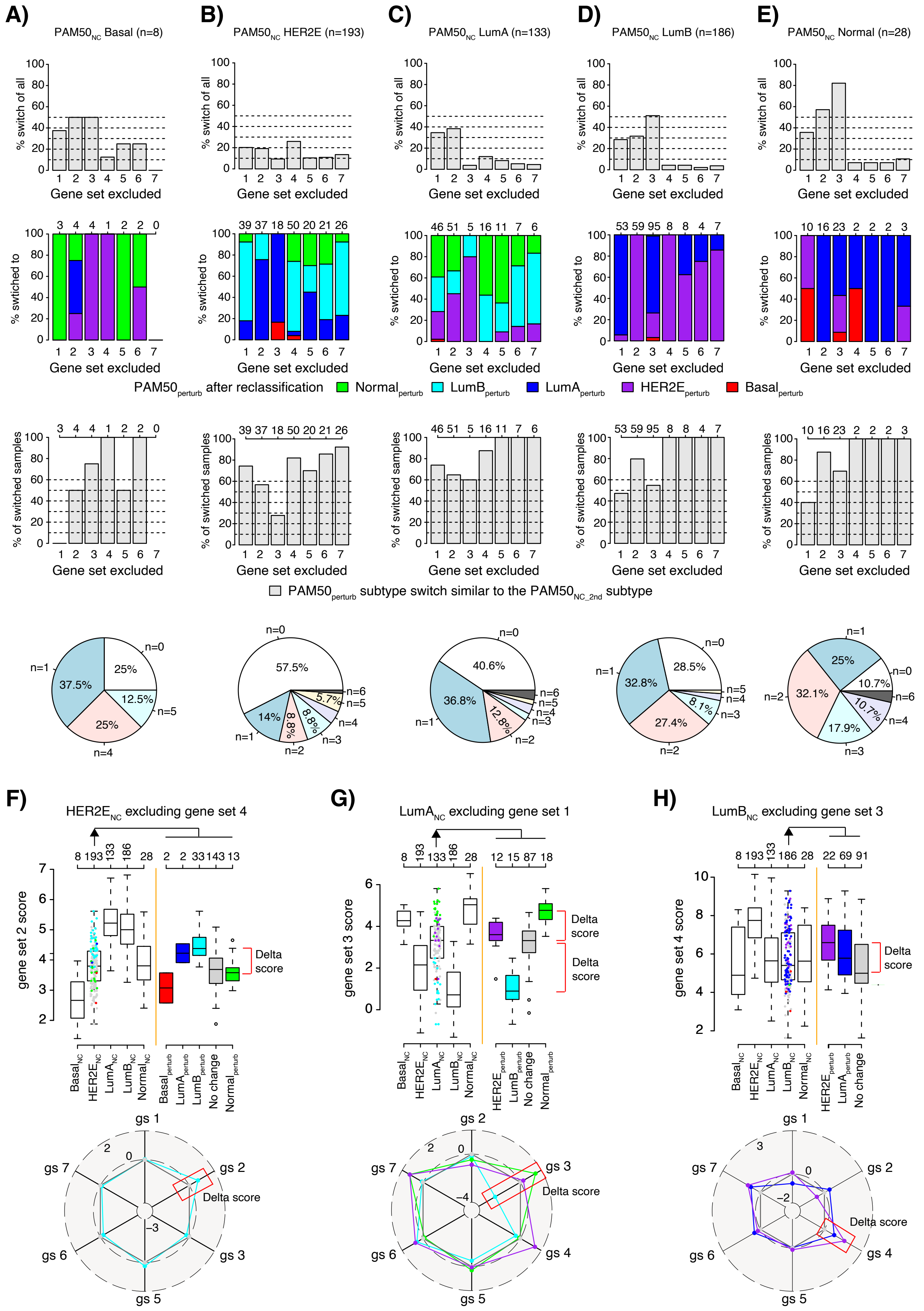

Supplementary Figure 4. PAM50perturb subtype switch effect of the leave-oneGeneCluster-out strategy in ERpHER2p disease. **A-E**) Top panels show the proportion of ERpHER2p tumors with a $PAM50_{perturb}$ subtype different from $PAM50_{NC}$, i.e., switching subtype, when excluding a specific gene set in *leave-oneGeneCluster-out* reclassification. Second level panels show the distribution of the $PAM50_{perturb}$ subtypes in tumors that switched in the top panel, number at top represents total number of samples that switched subtype. Third level panels show how many of the tumors that switched subtype after gene set exclusion that had a $PAM50_{perturb}$ subtype similar to the $PAM50_{NC\_2nd}$ subtype, number at top represents total number of samples that switched subtype. Bottom pie charts show the proportion of samples that switched n number of times across all gene set exclusions, e.g., size of n=6 equals the proportion of all tumors that had a $PAM50_{perturb}$ switch in 6 out of 7 gene set exclusions. $PAM50_{perturb}$ subtype switch was calculated in **A**) eight $Basal_{NC}$ tumors, **B**) 193 $HER2E_{NC}$ tumors, **C**) 133 $LumA_{NC}$ tumors, **D**) 186 $LumB_{NC}$ tumors, and **E**) 28 $Normal_{NC}$ tumors. **F**) Top panel: boxplots of gene set 2 scores for all ERpHER2p tumors stratified by $PAM50_{NC}$ subtypes (left part, representing original data), and for $HER2E_{NC}$ tumors stratified by the $PAM50_{perturb}$ subtype after exclusion of gene set 4 (right part, representing perturbed subtypes). The difference in median gene set 2 score for perturbed cases with a $HER2E_{perturb}$ subtype (No change, gray) versus tumors with a $LumB_{perturb}$ is illustrated. Bottom panel: Spider chart of all median gene set (gs 1-7) expression scores for $HER2E_{NC}$ tumors with a $LumB_{perturb}$ (n=33) subtype after exclusion of gene set 4 (gs4 excluded from the chart). Rings correspond to delta score values for a specific gene set centered to the no change tumors (ring value 0). The gs 2 delta score difference highlighted in the top boxplot is highlighted in the corresponding axis of the spider chart for the $LumB_{perturb}$ tumors (red box). **G**) Similar illustrations as in F for $LumA_{NC}$ ERpHER2p tumors after exclusion of gene set 1. Boxplots show gs 3 (basal keratins) scores, with the gs 3 delta score differences between $Normal_{perturb}$ (n=18), $LumB_{perturb}$ (n=15) versus $LumA_{perturb}$ (No change) highlighted also in the corresponding spider chart showing all gene sets. **H**) Similar illustrations as in F for $LumB_{NC}$ ERpHER2p tumors after exclusion of gene set 3. Boxplots show gs 4 (*ERBB2*/*GRB7*) scores, with the gs 4 delta score difference between $HER2E_{perturb}$ (n=22) and $LumB_{perturb}$ (No change) highlighted also in the corresponding spider chart showing all gene sets for $HER2E_{perturb}$ and $LumA_{perturb}$ tumors.

Boxplot elements correspond to: (i) center line = median, (ii) box limits = upper and lower quartiles, (iii) whiskers = 1.5x interquartile range.

Supplementary Figure 5. ERpHER2n

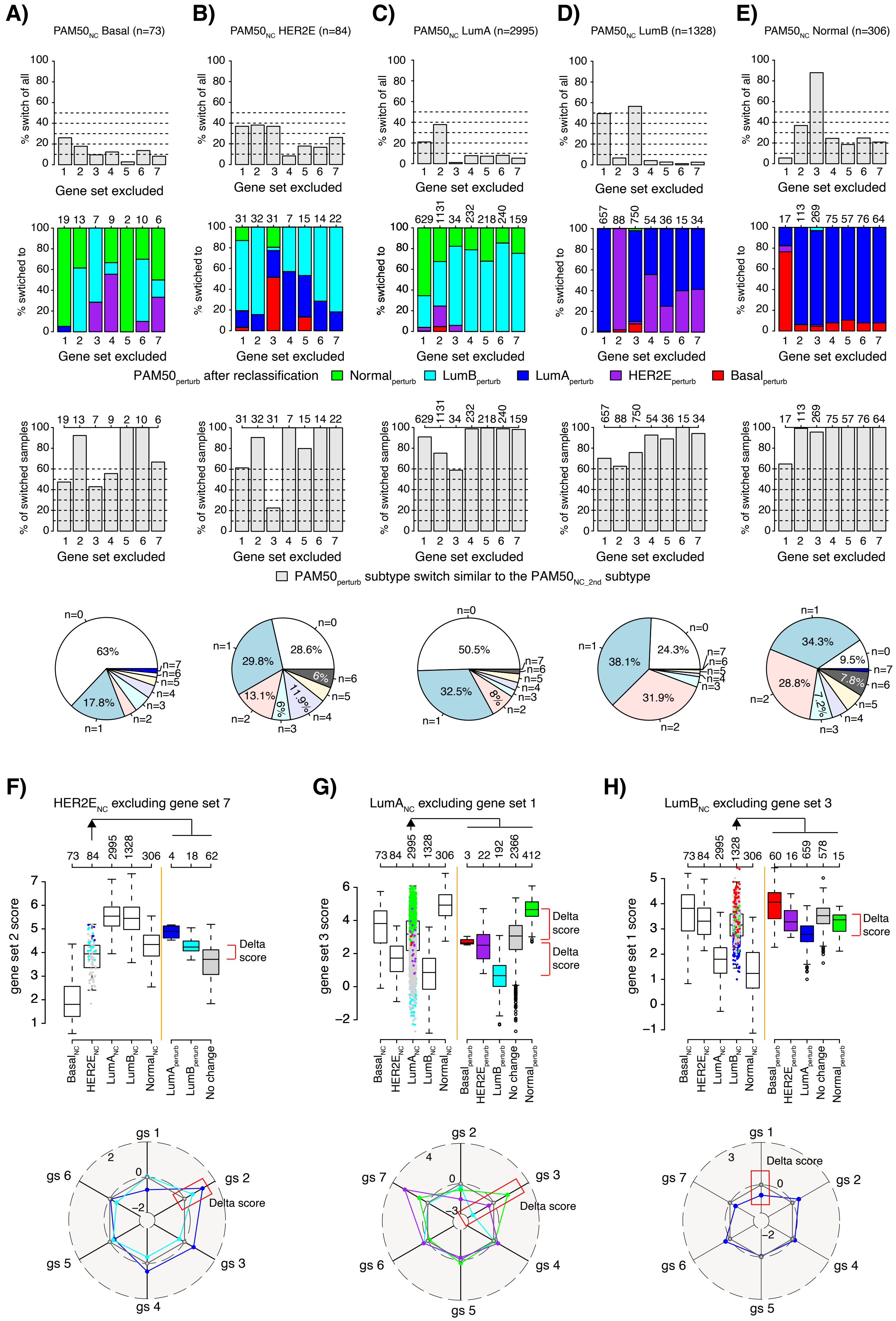

Supplementary Figure 5. PAM50perturb subtype switch effect of the leave-oneGeneCluster-out strategy in ERpHER2n disease. A-E) Top panels show the proportion of ERpHER2n tumors with a $PAM50_{perturb}$ subtype different from $PAM50_{NC}$, i.e., switching subtype, when excluding a specific gene set in *leave-oneGeneCluster-out* reclassification. Second level panels show the distribution of the $PAM50_{perturb}$ subtypes in tumors that switched in the top panel, number at top represents total number of samples that switched subtype. Third level panels show how many of the tumors that switched subtype after gene set exclusion that had a $PAM50_{perturb}$ subtype similar to the $PAM50_{NC\_2nd}$ subtype, number at top represents total number of samples that switched subtype. Bottom pie charts show the proportion of samples that switched n number of times across all gene set exclusions, e.g., size of n=6 equals the proportion of all tumors that had a $PAM50_{perturb}$ switch in 6 out of 7 gene set exclusions. $PAM50_{perturb}$ subtype switch was calculated in **A**) 73 $Basal_{NC}$ tumors, **B**) 84 $HER2E_{NC}$ tumors, **C**) 2995 $LumA_{NC}$ tumors, **D**) 1328 $LumB_{NC}$ tumors, and **E**) 306 $Normal_{NC}$ tumors. **F**) Top panel: boxplots of gene set 2 scores for all ERnHER2p tumors stratified by $PAM50_{NC}$ subtypes (left part, representing original data), and for $HER2E_{NC}$ tumors stratified by the $PAM50_{perturb}$ subtype after exclusion of gene set 7 (right part, representing perturbed subtypes). The differences in median gene set 2 score for perturbed cases with a $HER2E_{perturb}$ subtype (No change, gray) versus tumors with a $LumB_{perturb}$ or $LumA_{perturb}$ subtype are illustrated. Bottom panel: Spider chart of all median gene set (gs 1-7) expression scores for $HER2E_{NC}$ tumors with a $LumB_{perturb}$ (n=18) or $LumA_{perturb}$ (n=4) subtype after exclusion of gene set 7 (gs7 excluded from the chart). Rings correspond to delta score values for a specific gene set centered to the no change tumors (ring value 0). The gs 2 delta score difference highlighted in the top boxplot is highlighted in the corresponding axis of the spider chart for the $LumB_{perturb}$ and $LumA_{perturb}$ tumors (red box). **G**) Similar illustrations as in F for $LumA_{NC}$ tumors after exclusion of gene set 1. Boxplots show gs 3 (basal keratins) scores, with the gs 3 delta score differences between $Normal_{perturb}$ (n=412) and $LumB_{perturb}$ (n=192) versus $LumA_{perturb}$ (No change) highlighted also in the corresponding spider chart showing all gene sets ($Basal_{perturb}$ excluded). **H**) Similar illustrations as in F for $LumB_{NC}$ tumors after exclusion of gene set 3. Boxplots show gs 1 (proliferation) scores, with the gs 1 delta score difference between $LumA_{perturb}$ (n=659) and $LumB_{perturb}$ (No change) highlighted also in the corresponding spider chart showing all gene sets for $LumA_{perturb}$ tumors only.
Boxplot elements correspond to: (i) center line = median, (ii) box limits = upper and lower quartiles, (iii) whiskers = 1.5x interquartile range.

A) Subtyped as $Basal_{NC}$

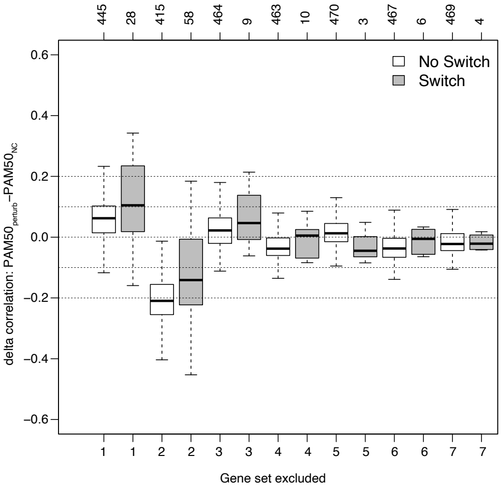

B) Subtyped as $HER2E_{NC}$

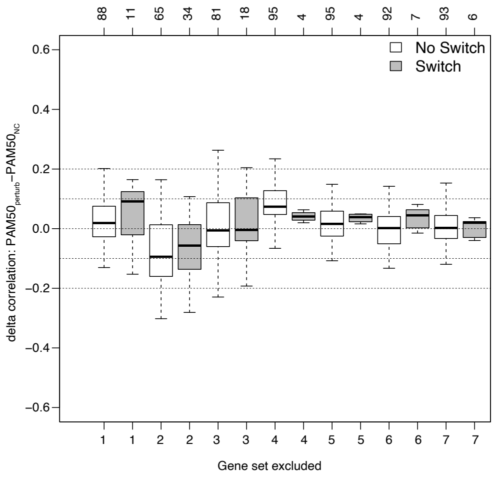

C) Subtyped as $LumA_{NC}$

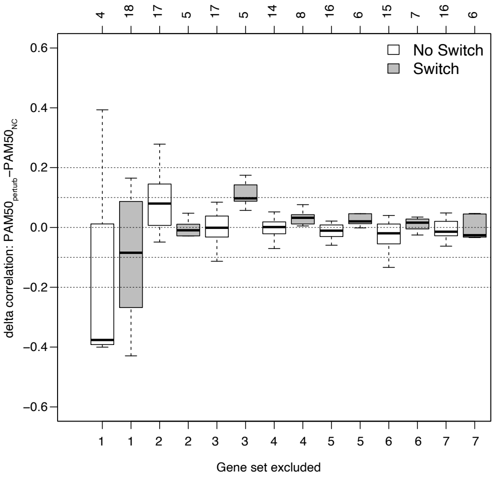

D) Subtyped as $LumB_{NC}$

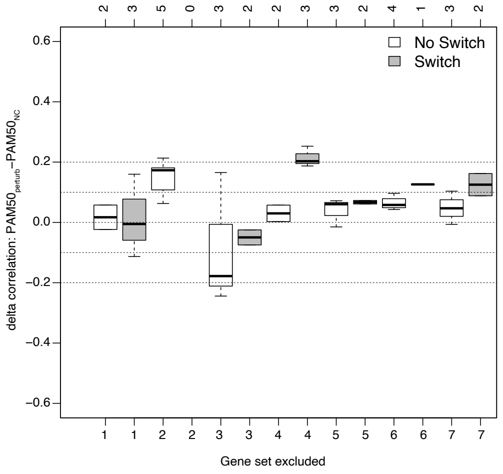

E) Subtyped as $Normal_{NC}$

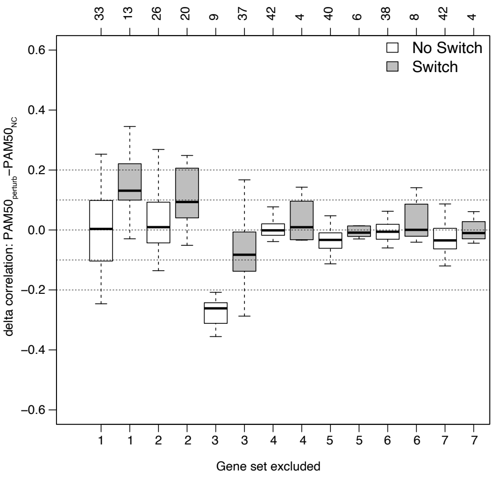

Supplementary Figure 6. Difference (delta-value) in subtype correlations between TNBC tumors stratified by their $PAM50_{NC}$ subtypes. The delta-value corresponds to the correlation for the $PAM50_{perturb}$ subtype after *leave-OneGeneCluster-out* reclassification minus the $PAM50_{NC}$ subtype correlation. White boxes represent samples where there were no switch in subtype after reclassification, i.e. , for instance $Basal_{NC}$ before and $Basal_{perturb}$ after reclassification. Grey boxes represent cases where a subtype switch occured, e.g., $Basal_{NC}$ before but $HER2E_{perturb}$ after reclassification based on exclusion of a specific gene set. (**A**) $Basal_{NC}$ tumors. (**B**) $HER2E_{NC}$ tumors. (**C**) $LumA_{NC}$ tumors. (**D**) $LumB_{NC}$ tumors. (**E**) $Normal_{NC}$ tumors.
Boxplot elements correspond to: (i) center line = median, (ii) box limits = upper and lower quartiles, (iii) whiskers = 1.5x interquartile range.

A) Subtyped as $Basal_{NC}$

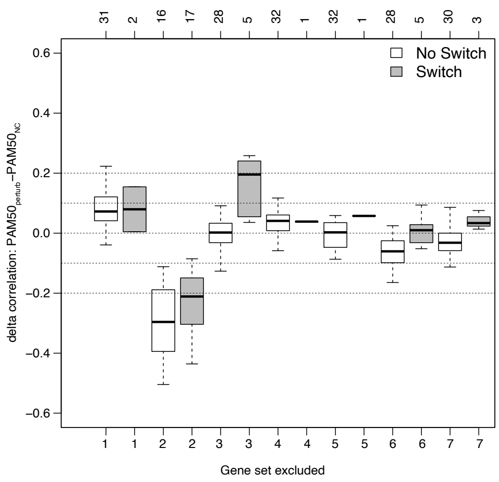

B) Subtyped as $HER2E_{NC}$

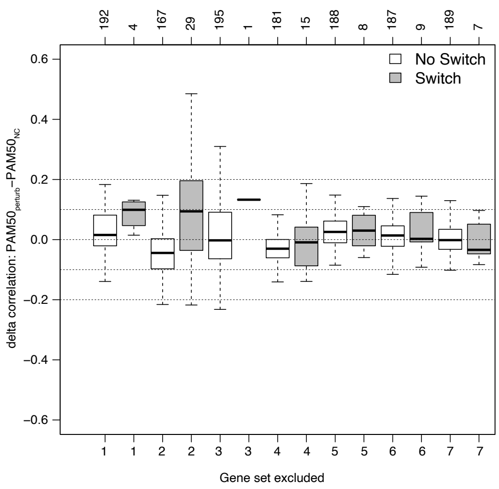

C) Subtyped as $LumA_{NC}$

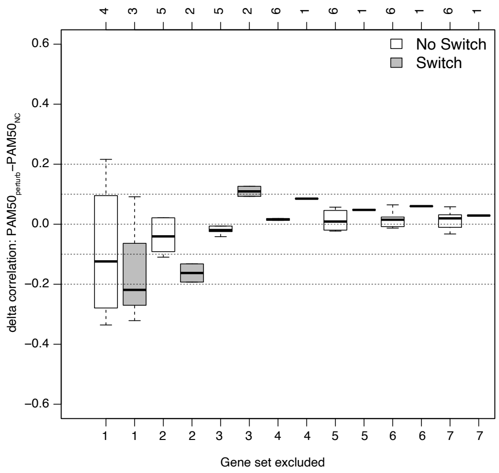

D) Subtyped as $LumB_{NC}$

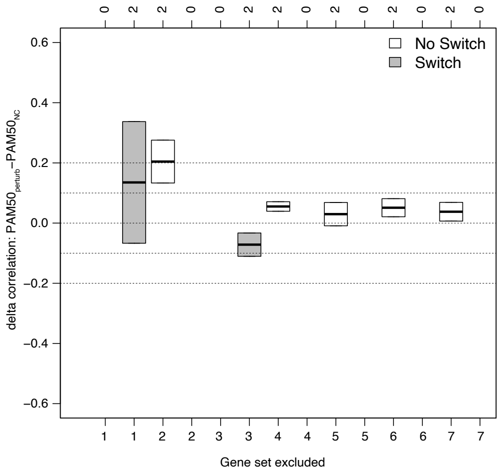

E) Subtyped as $Normal_{NC}$

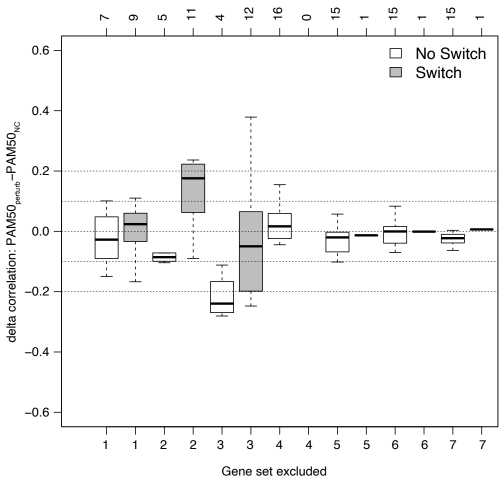

Supplementary Figure 7. Difference (delta-value) in subtype correlations between ERnHER2p tumors stratified by their $PAM50_{NC}$ subtypes. The delta-value corresponds to the correlation for the $PAM50_{perturb}$ subtype after *leave-OneGeneCluster-out* reclassification minus the $PAM50_{NC}$ subtype correlation. White boxes represent samples where there were no switch in subtype after reclassification, i.e. , for instance $Basal_{NC}$ before and $Basal_{perturb}$ after reclassification. Grey boxes represent cases where a subtype switch occured, e.g., $Basal_{NC}$ before but $HER2E_{perturb}$ after reclassification based on exclusion of a specific gene set. (**A**) $Basal_{NC}$ tumors. (**B**) $HER2E_{NC}$ tumors. (**C**) $LumA_{NC}$ tumors. (**D**) $LumB_{NC}$ tumors. (**E**) $Normal_{NC}$ tumors.

Boxplot elements correspond to: (i) center line = median, (ii) box limits = upper and lower quartiles, (iii) whiskers = 1.5x interquartile range.

A) Subtyped as $Basal_{NC}$

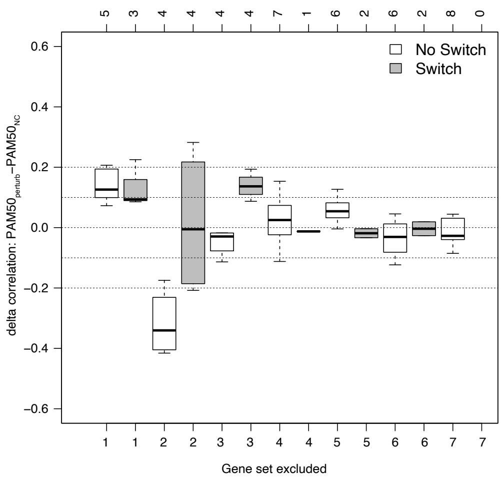

B) Subtyped as $HER2E_{NC}$

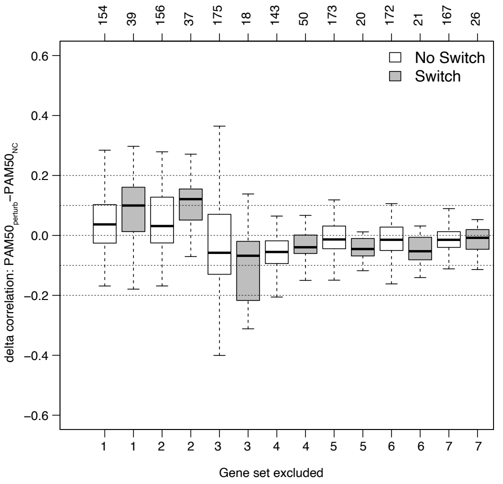

C) Subtyped as $LumA_{NC}$

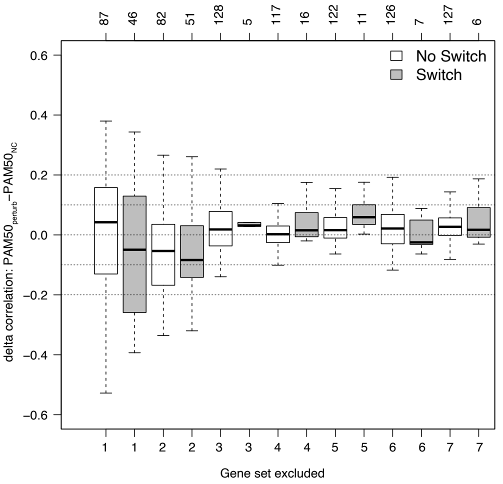

D) Subtyped as $LumB_{NC}$

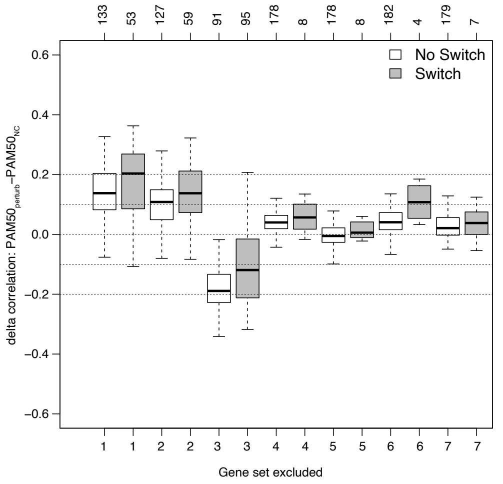

E) Subtyped as $Normal_{NC}$

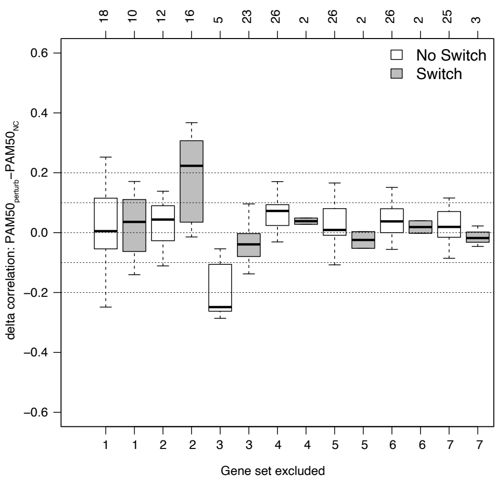

Supplementary Figure 8. Difference (delta-value) in subtype correlations between ERpHER2p tumors stratified by their $PAM50_{NC}$ subtypes. The delta-value corresponds to the correlation for the $PAM50_{perturb}$ subtype after *leave-OneGeneCluster-out* reclassification minus the $PAM50_{NC}$ subtype correlation. White boxes represent samples where there were no switch in subtype after reclassification, i.e. , for instance $Basal_{NC}$ before and $Basal_{perturb}$ after reclassification. Grey boxes represent cases where a subtype switch occured, e.g., $Basal_{NC}$ before but $HER2E_{perturb}$ after reclassification based on exclusion of a specific gene set. (**A**) $Basal_{NC}$ tumors. (**B**) $HER2E_{NC}$ tumors. (**C**) $LumA_{NC}$ tumors. (**D**) $LumB_{NC}$ tumors. (**E**) $Normal_{NC}$ tumors.

Boxplot elements correspond to: (i) center line = median, (ii) box limits = upper and lower quartiles, (iii) whiskers = 1.5x interquartile range.

A) Subtyped as $Basal_{NC}$

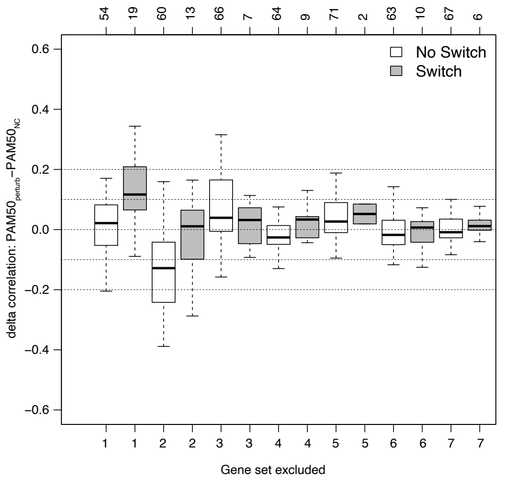

B) Subtyped as $HER2E_{NC}$

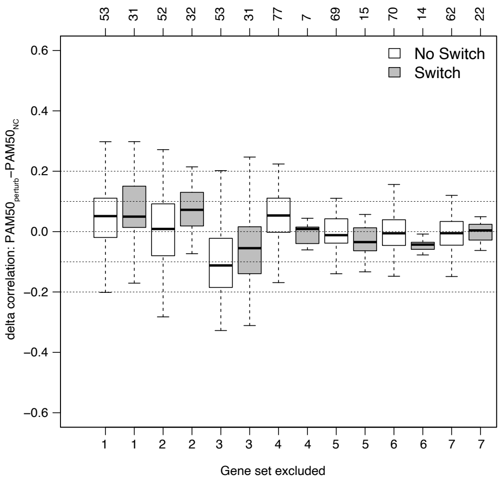

C) Subtyped as $LumA_{NC}$

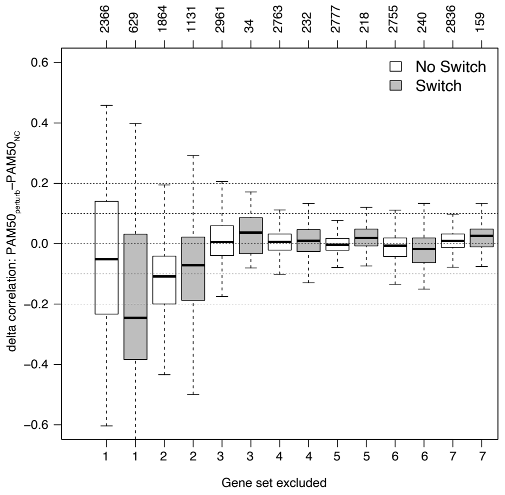

D) Subtyped as $LumB_{NC}$

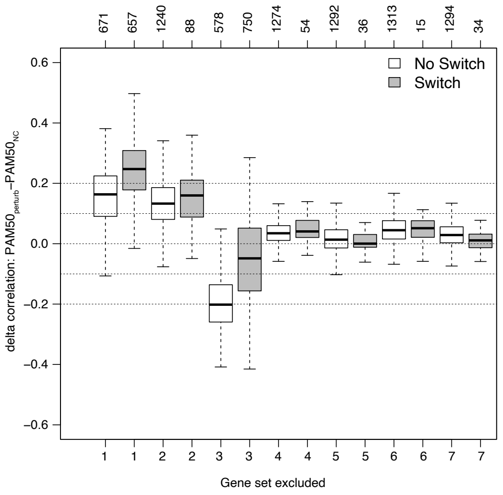

E) Subtyped as $Normal_{NC}$

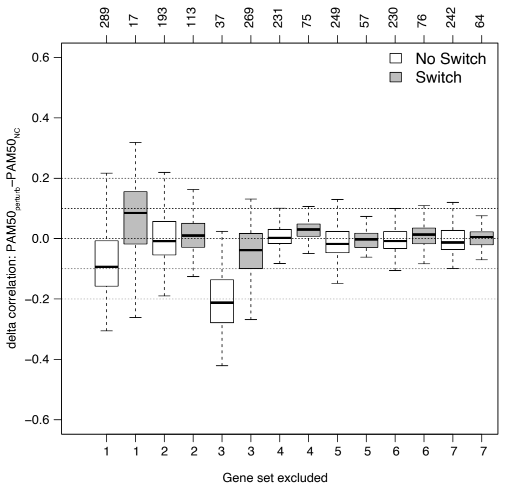

Supplementary Figure 9. Difference (delta-value) in subtype correlations between ERpHER2n tumors stratified by their $PAM50_{NC}$ subtypes. The delta-value corresponds to the correlation for the $PAM50_{perturb}$ subtype after *leave-OneGeneCluster-out* reclassification minus the $PAM50_{NC}$ subtype correlation. White boxes represent samples where there were no switch in subtype after reclassification, i.e. , for instance $Basal_{NC}$ before and $Basal_{perturb}$ after reclassification. Grey boxes represent cases where a subtype switch occured, e.g., $Basal_{NC}$ before but $HER2E_{perturb}$ after reclassification based on exclusion of a specific gene set. (**A**) $Basal_{NC}$ tumors. (**B**) $HER2E_{NC}$ tumors. (**C**) $LumA_{NC}$ tumors. (**D**) $LumB_{NC}$ tumors. (**E**) $Normal_{NC}$ tumors.

Boxplot elements correspond to: (i) center line = median, (ii) box limits = upper and lower quartiles, (iii) whiskers = 1.5x interquartile range.
