# Supplementary material for: Perturbation and stability of PAM50 subtyping in population-based primary invasive breast cancer
Source: NPJ Breast Cancer. 2023 Oct 19;9:83. doi: 10.1038/s41523-023-00589-0 (PMC10587090; doi:10.1038/s41523-023-00589-0)
Supplement: Supplementary file 4 — Reporting Summary [file 41523_2023_589_MOESM4_ESM.pdf]

nature portfolio

Corresponding author(s): Johan Staaf

Last updated by author(s): Aug 6, 2023

Reporting Summary

Nature Portfolio wishes to improve the reproducibility of the work that we publish. This form provides structure for consistency and transparency in reporting. For further information on Nature Portfolio policies, see our Editorial Policies and the Editorial Policy Checklist.

Statistics

For all statistical analyses, confirm that the following items are present in the figure legend, table legend, main text, or Methods section.

n/a	Confirmed	
☐	☒	The exact sample size (*n*) for each experimental group/condition, given as a discrete number and unit of measurement
☒	☐	A statement on whether measurements were taken from distinct samples or whether the same sample was measured repeatedly
☐	☒	The statistical test(s) used AND whether they are one- or two-sided<br>*Only common tests should be described solely by name; describe more complex techniques in the Methods section.*
☐	☒	A description of all covariates tested
☐	☒	A description of any assumptions or corrections, such as tests of normality and adjustment for multiple comparisons
☐	☒	A full description of the statistical parameters including central tendency (e.g. means) or other basic estimates (e.g. regression coefficient) AND variation (e.g. standard deviation) or associated estimates of uncertainty (e.g. confidence intervals)
☐	☒	For null hypothesis testing, the test statistic (e.g. *F*, *t*, *r*) with confidence intervals, effect sizes, degrees of freedom and *P* value noted<br>*Give P values as exact values whenever suitable.*
☒	☐	For Bayesian analysis, information on the choice of priors and Markov chain Monte Carlo settings
☒	☐	For hierarchical and complex designs, identification of the appropriate level for tests and full reporting of outcomes
☐	☒	Estimates of effect sizes (e.g. Cohen's *d*, Pearson's *r*), indicating how they were calculated

Our web collection on statistics for biologists contains articles on many of the points above.

Software and code

Policy information about availability of computer code

Data collection

References refer to the main manuscript.

Data collection
6233 patients diagnosed with primary invasive breast tumors and enrolled in the Sweden Cancerome Analysis Network – Breast (SCAN-B) study [23, 24] (ClinicalTrials.gov ID NCT02306096) from 2010 to 2018 with curated RNA sequencing data and complete clinicopathological and PAM50 data (specifically PAM50 classification as Basal, HER2E, LumA, LumB, or Normal, ER, PR, HER2, and nodal status, treatment indication, and patient follow-up) available in Staaf et al. [14] were included. The included cohort is hereafter referred to as SCAN-B. The 6233 patients comprise 93.6% of the 6660-sample early-stage follow-up cohort (one patient – one tumor RNA sequencing profile) defined in [14] from the total set of 9206 RNA sequencing profiles in [14]. Clinicopathological and molecular characteristics for the 6233 patients' tumors are detailed in Supplementary Table S1. Specific patient inclusion and exclusion criteria for the SCAN-B cohort are reported in the original publication [14]. Patients in this cohort have previously been shown to be representative of the underlying breast cancer population of the healthcare region in which they were enrolled [14, 22]. The PAM50 classification used in this study is based on the five-subtype system (Basal, HER2E, LumA, LumB, Normal) using the NC classification methodology reported in [14] (therein termed NCN). In the classification approach described in [14], each tumor is subtyped 100 times using 100 different reference sets for centering, resulting in 100 correlations to each PAM50 centroid from which a majority subtype vote is determined. The majority subtype is hereon referred to as a tumor's PAM50NC subtype (BasalNC, HER2ENC, LumANC, LumBNC, NormalNC). For calculation of a tumor's correlation to the best (PAM50NC) and second-best (PAM50NC_2nd) NC subtype, the average correlation per centroid of the 100 correlation values was used. This average value was also used to determine the PAM50NC_2nd subtype for a tumor. Patients were divided into four clinically relevant subgroups (with different therapy options) according to ER, PR, and HER2 status (p=positive, n=negative) available from clinical cancer registry: i) TNBC, ii) ERnHER2p, iii) ERpHER2p, and iv) ERpHERn.

Data analysis

All analyses were performed using open-source software such as Java and the R statistical language. Specific sources of code used in this study

Data analysis

for the SRIQ co-expressed gene clusters analysis can be found at https://github.com/StaafLab/SRIQ.

PPAM50 reclassification following a leave-oneGeneCluster-out strategy
PAM50 reclassification was performed by modifying the method outlined in [14] following a leave-oneGeneCluster-out strategy where individual SRIQ-derived gene sets were sequentially excluded. Three different gene matched data matrices were used: i) the PAM50 centroids from Parker et al. [7], ii) a matrix including 100 defined reference sets for gene centering as defined in [14], and iii) a matrix for the samples to be classified. The strategy was based on excluding one of the SRIQ-derived PAM50 gene clusters at a time from the centroid, reference set, and expression matrices, creating reduced gene matrices. For each sample NC classification using the remaining genes was performed 100 times using the 100 defined reference sets for gene centering as defined in [14]. The genes specifically included in the seven SRIQ core gene sets formed the background centroid gene content for these analyses. The resulting subtype, PAM50perturb, was called as Basalperturb, HER2Eperturb, LumAperturb, LumBperturb, and Normalperturb. A sample was called as having a subtype switch if the PAM50NC subtype was observed in [illegible]50% of the 100 PAM50perturb classifications and we used no minimum correlation cut-off for subtyping.

Statistical methods
All p-values reported are two-sided and were compared to a level of significance of 0.05 unless otherwise specified. Boxplot elements correspond to: (i) center line = median, (ii) box limits = upper and lower quartiles, (iii) whiskers = 1.5x interquartile range. Correlations were computed using Spearman correlation unless otherwise specified.

Survival analysis
Survival analyses were performed in R (v4.2.2) using the survival (v3.4.0) and survminer (v0.4.9) packages with overall survival (OS) and distant recurrence-free interval (DRFI) as primary endpoints obtained from [14]. Survival curves were estimated using the Kaplan-Meier method and compared using the log-rank test. Cox proportional hazard ratios were computed using the coxph function in R.

cBioPortal analyses
For gene-gene comparisons in TCGA breast cancers, we used the cBioPortal online tool (www.cbioportal.org). For correlation analyses, the option of log-transformed mRNA expression z-scores compared to the expression distribution of all samples (RNA Seq V2 RSEM) was used for 1082 tumors.

Functional pathway analyses
Functional analysis of the gene clusters was performed by pathway enrichment analysis using Enrichr (v3.1) [27, 28] accessing the KEGG pathway [29, 30] and Gene Ontology Consortium databases [31, 32] with an adjusted p-value cut-off of $p<0.05$,

Biological metagenes
Rank scores for eight biological metagenes reported by Fredlund et al. [15] was calculated as defined by Nacer et al. [33] and used in the study.

For manuscripts utilizing custom algorithms or software that are central to the research but not yet described in published literature, software must be made available to editors and reviewers. We strongly encourage code deposition in a community repository (e.g. GitHub). See the Nature Portfolio guidelines for submitting code & software for further information.

Data

Policy information about availability of data

All manuscripts must include a data availability statement. This statement should provide the following information, where applicable:

- Accession codes, unique identifiers, or web links for publicly available datasets
- A description of any restrictions on data availability
- For clinical datasets or third party data, please ensure that the statement adheres to our policy

Clinical, molecular, and processed RNA sequencing data (fragments per kilobase million, FPKM) were obtained from an open-access repository associated with the study by Staaf et al. [14] (An open access Mendeley repository).

In addition, Supplementary Table S1 includes all clinical and molecular data used for analyses in the current manuscript for the specific samples.

Research involving human participants, their data, or biological material

Policy information about studies with human participants or human data. See also policy information about sex, gender (identity/presentation), and sexual orientation and race, ethnicity and racism.

Reporting on sex and gender	Not relevant for the study. No sub analyses performed.
Reporting on race, ethnicity, or other socially relevant groupings	Not relevant for the study No sub analyses performed.
Population characteristics	Population characteristics are described in Staaf et al 2022 (PMID:35974007) from which publicly available data was used for this study.
Recruitment	Recruitment is described in Staaf et al 2022 (PMID:35974007) from which publicly available data was used for this study.
Ethics oversight	Regional Ethical Review Board in Lund, Sweden, governed by the Swedish Ethical Review Authority, Box 2110, 750 02 Uppsala, Sweden.

Note that full information on the approval of the study protocol must also be provided in the manuscript.

Field-specific reporting

Please select the one below that is the best fit for your research. If you are not sure, read the appropriate sections before making your selection.

☒ Life sciences ☐ Behavioural & social sciences ☐ Ecological, evolutionary & environmental sciences

For a reference copy of the document with all sections, see nature.com/documents/nr-reporting-summary-flat.pdf

Life sciences study design

All studies must disclose on these points even when the disclosure is negative.

Sample size

This study used available public data from Staaf et al 2022 (PMID:35974007). We only included patients with full clinical data needed to group patients into TNBC, ERnHER2p, ERpHER2p, and ERpHER2p groups as outlined in the manuscript.

Data exclusions

Based on public data from Staaf et al 2022 (PMID:35974007):
6233 patients diagnosed with primary invasive breast tumors and enrolled in the Sweden Cancerome Analysis Network – Breast (SCAN-B) study [23, 24] (ClinicalTrials.gov ID NCT02306096) from 2010 to 2018 with curated RNA sequencing data and complete clinicopathological and PAM50 data (specifically PAM50 classification as Basal, HER2E, LumA, LumB, or Normal, ER, PR, HER2, and nodal status, treatment indication, and patient follow-up) available in Staaf et al. [14] were included.

We only included patients with full clinical data needed to group patients into TNBC, ERnHER2p, ERpHER2p, and ERpHER2p groups as outlined in the manuscript.

Replication

RNAsequencing was not replicated if quality thresholds were met as outlined in Staaf et al 2022 (PMID:35974007) from which publicly available data was used for this study. To note, this study only used available in silico data deposited in an open access repository.

Randomization

Not relevant for this study.

Blinding

Not relevant for this study.

Reporting for specific materials, systems and methods

We require information from authors about some types of materials, experimental systems and methods used in many studies. Here, indicate whether each material, system or method listed is relevant to your study. If you are not sure if a list item applies to your research, read the appropriate section before selecting a response.

Materials & experimental systems

n/a	Involved in the study	
☒	☐	Antibodies
☒	☐	Eukaryotic cell lines
☒	☐	Palaeontology and archaeology
☒	☐	Animals and other organisms
☐	☒	Clinical data
☒	☐	Dual use research of concern
☒	☐	Plants

Methods

n/a	Involved in the study	
☒	☐	ChIP-seq
☒	☐	Flow cytometry
☒	☐	MRI-based neuroimaging

Clinical data

Policy information about clinical studies
All manuscripts should comply with the ICMJE guidelines for publication of clinical research and a completed CONSORT checklist must be included with all submissions.

Clinical trial registration

NCT02306096

Study protocol

SCAN-B is a prospective observational study. No intervention is performed.

Data collection

Data for this study was taken from a public repository associated with Staaf et al 2022 (PMID:35974007)

Outcomes

Clinical outcomes are defined in the original study by Staaf et al 2022 (PMID:35974007) from which we obtained publicly available data.
